# Intermediary liability and trade in follow-on innovation

**DOI:** 10.1007/s10824-023-09470-1

**Published:** 2023-02-12

**Authors:** Alexander Cuntz, Matthias Sahli

**Affiliations:** 1 Department for Economics and Data Analytics, World Intellectual Property Organization, 34, chemin des Colombettes, CH-1211 Geneva, Switzerland; 2 Institute of Economic Research, University of Neuchâtel, A.-L. Breguet 2, CH-2000 Neuchâtel, Switzerland

**Keywords:** Figurative arts, Copyright, Intermediary liability, Creative reuse, Appropriation Art, Auctions, Hedonic price models, Differences-in-differences, O31, O34, Z11

## Abstract

Intellectual property rights have changed the market value and direction of artistic innovation throughout art history, in particular when new creations built on the art of predecessors. In this paper, we test how changes in legal frameworks and litigation risks affected market value and commercial trade around artistic reuses in the figurative arts and the ‘Appropriation Art’ movement in particular. Appropriation artists borrow images from different sources and incorporate them into new, derivative works of art. By doing so, they risk infringing copyright but also put auction trade and artwork availability at litigation risk as liability can extend to market intermediaries, such as auction houses, museums, or galleries. Using a differences-in-differences model and large-scale online data, we investigate the causal impact of the prominent *Cariou v. Prince* U.S. higher court decision on intermediary trade and the availability of artworks on sale in the Appropriation Art. As an exogenous shock, this decision changed the perceived litigation risk for market intermediaries around what constitutes fair use. Following the court decision, we find a temporary decline in the total number of global auctions in the Appropriation Art, a lower sales probability of these artworks, and a relocation of related auctions to non-U.S. houses.

## Introduction

Recent research has helped to understand artistic innovation and creativity over an artist’s career (Galenson, 2010). Many innovations in the arts are cumulative, with new artists being influenced by their predecessors and existing culture (Irvin, 2005; Schaumann, 2015). Such an evolution goes back to Renaissance art, when artists were inspired by classical art and developed sequential artistic innovations building on the achievements of each other. And it is even more evident in contemporary art. In recent times, the Appropriation Art movement is a prominent example of cumulative creativity and innovation (Ames, 1993; Evans, 2009; Landes, 2000; Welchman, 2013). Since the 1980s, appropriation artists have been ‘deconstructing and recontextualizing’ pre-existing materials by incorporating photographic sources, advertising materials and other types of artistic work into their new derivative artworks (Evans, 2009). By incorporating pre-existing material, they risk infringing the rights of other artists and rights holders. Copying in general has become a central subject of contemporary art because of the shifts in both the art itself and the available technology (Adler, 2016). This and many other examples exemplify the process of imitation and innovation in the figurative arts, which has played a significant role in the development of various art movements and styles. The economics of art history focuses on how market factors and institutional changes affect the creation and the trade of artworks. Dealers and collectors in art markets recognize that the most innovative art becomes the most valuable over time, with many novel uses of old genres and the creation of some new ones in the art of the twentieth century (Galenson, 2009). Changes in intellectual property rules may have also affected the development of various art movements and styles throughout history, as laws impacted the production and diffusion of new art. The question of how changes in legal frameworks and litigation risks affected market value and commercial trade around artistic reuses in the Appropriation Art is the main focus of this paper.

Intellectual property rights impact how much artists will create and can build on the work of their predecessors. Copyright laws balance economic incentives for both original and follow-on creators, but also provide incentives for commercialization and sales to market intermediaries and traders. Most countries grant artists exclusive rights to their work for a certain period of time. Under moderate copyright terms (copyright length), laws can generate incentives to create new original work, as documented in pioneering research on Italian opera under the Napoleonic Empire (Giorcelli & Moser, 2020). However, this also means that other artists must obtain permission or a license to reuse or build upon the work of their predecessors and may have to pay fees or royalties. Broader terms (copyright breadth) can impede cumulative creativity and limit follow-on innovation in various industries, including recorded music, book publishing, scientific production, and knowledge reuse on Wikipedia (Biasi & Moser, 2016; Nagaraj, 2016; Reimers, 2019; Watson, 2017a; Watson et al., 2022).

In recent years, the Appropriation Art movement has become a central battleground for copyright infringement cases (Adler, 2018). Not only artists, but galleries, auction houses, and museums alike have been held liable for infringing rights when reusing, selling, or exhibiting these works in art markets. Our research uses hedonic price models, a difference-in-differences design, and large-scale data from online services to quantify the impact of the prominent *Cariou v. Prince* U.S. court decision and changes in perceived fair use rules on the level of trade and commercial availability in Appropriation Art. In this defining case of Appropriation Art (Sarmiento & Haaften-Schick, 2013), the photographer Patrick Cariou sued the well-known appropriation artists Richard Prince and the Gagosian Gallery promoting the latter artist for copyright infringement, arguing that fair use did not apply to Prince’s follow-on artwork. As an exogenous shock to the art market, the 2013 court decision ultimately extended infringement liability to market intermediaries (Gagosian Gallery), thereby increasing legal uncertainty in the auction market. We find that the total number of global auctions declined following the decision and that some trade in the Appropriation Arts temporarily relocated to jurisdictions outside the U.S. due to concerns over contributory liability and legal uncertainty. The sales probability of artworks listed in auctions also decreased. Effects on auction trade and curation continue to hold after controlling for superstar artists.

This paper contributes to the understanding of how market institutions such as copyright affect artistic innovation and cumulative creativity in figurative arts. It adds to the individual-level determinants and greater artistic freedom in competitive markets for contemporary art discussed in the economic art history literature (Galenson, 2010). And, it confirms the effect of broader laws on cumulative creativity and follow-on innovation found in previous research (Biasi & Moser, 2016; Nagaraj, 2016; Reimers, 2019; Watson, 2017a; Watson et al., 2022). As we can show in this paper, under broader terms of copyright protection, extended liability rules can change the relative market value and growth potential of certain art movements and appropriating styles that rely heavily on older work. This in turn affects the general direction of the figurative arts and trade in those art movements. Some production and follow-on innovation with greater exposure to legal risk are likely to be redirected to alternative and lower-risk art genres and investment fields that rely less on the art of predecessors.

Second, the research shows that the impact of legal uncertainty in the copyright framework affects and ‘trickles down’ on market intermediaries such as auction houses and galleries. Accordingly, causal findings corroborate the basic argument developed in the theoretical literature (Landes, 2000; Wu, 2004). Similar to the direct effect of broader copyrights on reuses (Green & Scotchmer, 1995; Murray & O’Mahony, 2007; Scotchmer, 1991), legal uncertainty around indirect liability rules impedes commercial trade and limits curation around follow-on innovation (Landes & Lichtman, 2003). This is important to note for the creative and cultural sectors, as intermediaries such as museums, galleries, or auction houses are important gatekeepers in the art market.

Third, research results contribute to the larger debate about how the (complementary) fair use doctrine under U.S. copyright law compares to (only) closed list of copyright exceptions in other countries, and whether fair use creates legal uncertainty because of its vague, standard-like nature (Beebe, 2008; Sag, 2012). In this paper, we provide first-hand quantitative evidence on the actual costs and market charges for imposing indirect liability on intermediaries within the fair use doctrine. In this way, our findings also add to the generic law and economics literature on resource allocation and transaction costs with its strong focus on theory development (Coase, 1960; Calabresi, 1960; Posner, 1972; Depoorter & Parisi, 2002a; Marciano, 2012).

Fourth, previous studies have documented how differences in national copyright laws can push trade to foreign jurisdictions as well as the broader economic effect of resale rights (Banternghansa & Graddy, 2011; Ginsburgh et al., 2005; Ginsburgh, 2005; Watt et al., 2014). In a similar vein, Landes and Lichtman (2003) argue that indirect liability creates incentives for online music platforms to shift services to jurisdictions that offer them more favorable rules. Our findings show that legal uncertainty around the fair-use doctrine, as reflected in U.S. copyright judicial decisions, can force the relocation of auction sales to foreign jurisdictions with less uncertainty. To the best of our knowledge, this is the first study to document such an effect.

The paper structures as follows. Section 2 provides an overview of the economic literature and some legal background on the reuse of images in the visual arts. Section 3 presents the data and the empirical framework. Section 4 gives results and robustness checks. Sections 5 and 6 discuss policy implications and conclude.

## Previous research and legal background

### Literature review

While quantitative studies in copyright economics focus on creative and cultural industries such as books, music, and movies (Adermon & Liang, 2010; Aguiar & Waldfogel, 2018; Giorcelli & Moser, 2020; Reimers, 2019; Towse, 2017), relatively little research has been done on the scope of copyright and on the visual arts sector.^1^ The role of copyright in the visual arts differs significantly from other sectors. First, there is likely lower demand for copies of visual artworks. Even at low prices, copies would be poor substitutes for original works and, traditionally, there is a limited market for print-multiples (Becker et al., 2000; Buccafusco et al., 2017). Second, there is a high premium value attached to the original artwork and ‘authenticity’ in general (Watt et al., 2014). In a similar vein, Landes and Levine (2006) argue that the general economic case for copyright protection is weakened for unique works such as paintings since the artist’s main income source is the first sale of the original work (in the absence of resale rights). Overall, for several reasons being copied seems less of a threat to visual artist’s work and the appropriation of value than it is for writers, composers, or performers in other sectors (Landes & Levine, 2006).

Still, there is an area of the visual arts where copyright laws play a decisive role, namely, in *Appropriation Art*, where copying others’ work is the modus operandi (Evans, 2009).^2^ Therefore, rules on the scope of copyright also will need to coordinate between different generations of creators and balance the sharing of economic value over time when their work is ‘sequential’ in nature. There is an argument in the literature that copyright is sometimes overreaching in the scope it provides to original creators compared to their successors (Buccafusco et al., 2017). National and international laws and legal traditions treat these reuses of works differently, also in the visual arts. For example, in certain jurisdictions, moral rights enshrined in copyright frameworks might require successive artists to credit and have their reuses of (selected) original works ‘authorized’ ex-ante, and also have follow-on creations reviewed and approved by original creators. Practically, these grant the latter a veto option toward commercialization of the ‘derivative’ work, which aims to ensure the ‘integrity’ of the original work and its creator. In other jurisdictions, ‘fair use’ limits copyright protection and allows for unauthorized copying in circumstances that roughly try to adapt to economic efficiencies (Landes & Posner, 1989). It is worth noting in this context that copyright limitations in other jurisdictions may not be as ‘favorable’ to Appropriation Art as the U.S. fair use defense can be (Geiger, 2020; Lucas & Ginsburg, 2016). In particular, the latter doctrine has included in the U.S. the concept of ‘transformativeness’ which should protect creativity by allowing to some extent new creative artwork building on pre-existing works [see Sect. 2.2 ‘Copyright Rules and the Reuse of Images’ and Adler (2016)], based on the idea that this can safeguard artistic freedom and space to operate.

Landes and Lichtman (2003) were first to discuss the economics of indirect liability for infringement. From the perspective of the law, indirect (or contributory) liability is an alternative mechanism to rights enforcement. Direct infringers often rely on technology, services, and venues provided by other third parties and these parties can be held liable for ‘facilitating’ infringement even when most of their sales are legitimate. As a consequence of indirect liability, for example, online hosting platforms might be asked to invest in and implement new enforcement and filtering technologies to comply with laws. Whether or not an indirect liability of intermediaries can provide a more effective route of enforcement than enforcing rights with direct infringers is not clear *ex-ante* and needs assessment.^3^

Landes and Lichtman (2003) argue that the proper scope for indirect liability can be determined by weighing its costs and benefits against those associated with other plausible mechanisms for rewarding creators which replicates their general idea in other papers to fully account for the cost of maintaining the copyright system when defining its boundaries. Our research makes an important contribution to that discussion by providing quantitative evidence on some of the actual costs and market charges for imposing indirect liability on intermediaries. Because they can also be held liable, they might be less willing to curate and offer reuses that are possibly infringing goods to buyers.

Moreover, this research contributes to the debate on the appropriate balance of copyright in cumulative creativity, suggesting that broad copyrights might impede reuse and follow-on innovation (Biasi & Moser, 2016; Nagaraj, 2016; Reimers, 2019; Watson, 2017a; Watson et al., 2022). Several of the underlying economic mechanisms around creative reuse deserve attention here. First, copyright incentivizes original creators to consider the additional value of productive reuses or ‘remixes’ downstream, without cannibalizing their own returns (Watson, 2017b). In this way, well-balanced rights might help expand the total output of original works and remixes (Gans, 2015; McLeod & DiCola, 2011).

Second, it may take the establishment of fair use rules or pre-set compensation schemes to overcome strategic hold-up and transaction costs problems around the efficient licensing of such reuses, in particular for productive rather than reproductive ones (Landes & Posner, 2003, 2009; Posner, 2004).^4^ Put differently, the economic efficiency around fair use can include circumstances with high transaction costs in which the benefits to the copier are higher than the costs of negotiation with the upstream right holder (Landes & Levine, 2006). For example, Watson (2017b) argues that hip-hop artists reusing pre-existing recordings (i.e., digital sampling in music) spend excess information and search costs before they can enter negotiations over licenses with the relevant right holders. Thus, ex-post licensing together with sunk cost creates hold-up inefficiencies for potential reuses.

Third, when downstream artists invest in creating a derivative work based on multiple sources, complementarities arise between original works (Watson, 2017b). As a result, right holders might individually charge higher licensing fees above the optimal level, so-called ‘royalty stacking’ increasing the cost of downstream reuses, an issue previously discussed in the context of patents (Farrell et al., 2007). In yet another paper, Watson (2017a) uses a matched-sample difference-in-differences design and reproductive ‘reuse’ instances as a quasi-exogenous shock to the music streaming of the original/underlying song. Using Spotify’s similarity algorithm, he finds that downstream reuses exhibit positive demand effects via advertising on the streaming of original songs, thereby moderating ex-post competition from reusing songs. These effects are larger for less prominent artists and first-time reuses of original songs. Both papers (Watson, 2017a, 2017b) serve as an inspiration for the research design and methodological approach developed in this paper.

Reuses in Appropriation Art in particular are often productive rather than reproductive in nature. Hence, it is unlikely that, in this particular field of the visual arts, downstream works do more harm to original works and will cannibalize their anticipated licensing revenues as in other sectors (Landes, 2000; Martin, 1985; Watson, 2017a). Rather it seems, based on the literature on cumulative creativity and copyright effects, artistic freedom to operate and reuse practices in the Appropriation Arts could be affected by changes in legal frameworks, including judicial decisions. A similar situation might well apply to market intermediaries that are facing higher litigation and liability risks when trading possibly infringing reuses compared to other types of original artworks. Ultimately, this is the empirical question we want to address in this paper. In the following section, we briefly summarize basic legal concepts, in particular U.S. fair use rules as applicable in the visual arts sector.

### Copyright rules and the reuse of images

As argued above, an important affirmative defense in U.S. copyright law is the fair use doctrine (Landes & Levine, 2006). Fair use allows for unauthorized copying in circumstances that are roughly consistent with promoting economic efficiency (Landes, 2000). That said, fair use in these terms is fair when ‘the cost of transacting with the copyright owner over permission to use the copyrighted work would exceed the benefits of transacting’ [(Posner, 1992), p.69, and (Landes & Posner, 1989)]. In broad terms, the fair use codified in section 107 of the U.S. Copyright Act allows for fair use and reproduction of a copyrighted work for purposes such as criticism, comment, teaching, research, or news reporting and provides the four factors to be used in determining whether a particular use made of a work is a fair use [for an application to the visual arts, see (Adler, 2016, 2018; Schaumann, 2015; Whitaker, 2019)].

Other legal systems also include rules prescribing exceptions and limitations for the scope of copyright protection, such as the British right of ‘parody,’ known as ‘fair dealing,’ or the French ‘droit de citation’ (Depoorter & Parisi, 2002b). Also, Israel and South Korea currently have implemented limited fair use provisions (Watson, 2017a). Generally, scholars on fair use argue that the doctrinal complexity and legal uncertainty can result in higher intellectual property enforcement costs (Depoorter et al., 2019). The adaption of the open-ended standard of the copyright doctrine to technological advances is discussed in Depoorter (2008) and Menell (2002). Furthermore, Liu (2019) provides an empirical study of transformative fair use jurisprudence for all types of copyrighted works in the U.S.

Certainly, when it comes to copyright law in the visual arts, the fair use doctrine is the subject of a controversial debate based on U.S. copyright jurisprudence. Appropriation Art has been challenged in the U.S. courts many times as an infringement of copyright of the ‘appropriated’ works (Agarwal, 2017).^5^ As a landmark case (Adler, 2018) in fair use, scholars point out the decision of *Cariou v. Prince* in 2013. The artist Richard Prince *and* the Gagosian Gallery as the market intermediary were sued for copyright infringement by the photographer Cariou for incorporating altered versions of his photographs into Prince’s series of artwork [see, e.g., (Francis, 2014)]. The lower court ruled in favor of the upstream photographer Cariou and found that the whole artwork series was infringing the copyright in the original content. Interestingly, not only was Prince held liable for copyright infringement but the Gagosian Gallery was also found to have ‘vicarious and contributory’ liability (Adler, 2016). The court stated that *‘the Gagosian Defendants had the right and ability to ensure that Prince obtained licenses to use the Photos before they made Prince’s paintings available for sale’ *.^6^ As an exogenous shock in the art market, the Second Circuit found that fair use would not be applicable to the entire artwork series and created high legal uncertainty (Sarmiento & Haaften-Schick, 2013) on the ‘vicarious and contributory’ liability for the market intermediaries, as we argue.

The higher court decision refined the fair use test in important ways that made it, arguably, more complicated — at least in the short term—to predict whether Appropriation Art is a copyright violation of the original artwork or not. Accordingly, it made the law even less predictable for artists and everyone involved in the trade and curation of these works (Adler, 2018). Moreover, the decision undermined the importance of other fair use factors with its unclear boundaries and an increased emphasis on transformation (Agarwal, 2017). The mere possibility of fair use litigation would now threaten away artists and their intermediaries and so ‘fair use is broken’ (Whitaker, 2019). In Adler (2016), the decision is described as the most urgent art law case, and it has brought a state of ‘panic’ to the art world. What is more, the court decision also has not been well-received in the art scene, for instance, the New York Times had titled ‘one of the most closely watched copyright cases to rattle the world of fine art’ and that it ‘set off alarm bells [...] in museums showing contemporary art’ (NewYorkTimes, 2011).^7^ In any case, this controversial and prominent court decision in 2013 has become the new boundary-drawing case of copyright infringement and Appropriation Art and it substantially increased legal uncertainty and litigation risks (Sarmiento & Haaften-Schick, 2013).

We argue that there was a somewhat more complex market response as the decision is said to have increased overall legal uncertainty for downstream artists and derivative works on sale and in exhibitions. Hence, the role of fair use in the visual arts and how it affected behavior on markets, ultimately, is an empirical question we want to address. Accordingly, we can motivate the focus on trade intermediaries such as auction houses by the legal uncertainty these decisions induced in the legal framework and the broad publicity they received.

## Data and empirical framework

### Data and matching

The unique dataset is compiled from the ‘Art Genome Project’ and its hosting service Artsy (2019). The online matchmaker brings together reputable galleries, auction houses, and art buyers globally. Next to hosting thousands of artwork for sale online, it also presents educational material and, provided its mere scale, also serves as a ‘reference’ catalog on visual arts history to users. Its technology builds on a growing database containing more than 50’000 artists. The ‘Art Genome Project’ classifies, connects, and characterizes each of these artists with currently over 1’000 characteristics, so-called ‘genomes’ (Artsy, 2019). In this way, the ‘Art Genome Project’ is a classification system, manually conducted by art historians and data scientists.

As a first step, we identify and attribute artists to the field of Appropriation Art, based on the artist-level ‘appropriation’ genome recorded in the Artsy data. API queries result in 1’901 unique artists and their biographies, including information on place and year of birth and death, nationality, and current work locations.^8^ As a second step, we survey the complete list of genomes and identify other genomes from the Art Genome Project indicating other types of reuse practices.^9^ This provides additional information on other reusing genomes (other than the appropriation genome collected in the first stage) for the initial set of appropriation artists, i.e., up to eight different ‘genomes’ around reuse practices can now be assigned to an individual artist of the first query. To create our control group of artists, we retrieve information on similar artists based on Artsy’s similarity algorithm and the Art-Genome-Project nearest neighbor graphs (i.e., related artists, however, excluded all artists with one or more appropriation-close genome-information).^10^ This gives us another 2,362 artists with a similar set of genomes as the one recorded for the initial set of appropriation artists. To sum up, our Artsy dataset contains 4263 unique artists meeting one or more criteria (i) a tight definition of appropriation based on a single ‘appropriation’ genome, (ii) the first criteria plus a wider definition of appropriation based on multiple genomes associated with different reuse practices and (iii) being similar based on a vector calculation across all Artsy genomes, however, not meeting one of the first two criteria (control group).

Prominent artists in our data associated with the ‘appropriation genome’ include Jeff Koons, Elaine Sturtevant, Richard Prince, Andy Warhol, Roy Lichtenstein, Banksy, Damien Hirst, and Louise Lawler, among others. For well-known similar artists, the Artsy algorithm identifies, for example, Andreas Gursky, Claes Oldenburg, Pablo Picasso, William Scott, and John Chamberlain. Less well-known artists in our similar (appropriation) group include Melissa Scott-Miller, John Sonsini, Mira Schor, Wanda Pimentel, and Shao Yinong (Steven Gagnon, Brad Faine, Joe Black, Lara Baladi, and Nancy Chunn). In general, ‘similar’ artists are not closely associated with appropriation and related artistic practices, but they will have very similar characteristics in terms of the various other genomes (Artsy, 2021). So, for example, a large set of genomes on Artsy classifies artists and artworks according to their ‘subject matter’ which alone includes more than 200 different genomes (political events, city scenes, figures from the back, body parts, bathers, decay, etc.). Similarly, Artsy’s category ‘materials’ carries more than 40 genomes (wood, aluminum, glass, gold, ivory, paper, etc.), ‘medium and techniques’ carries close to 200 genomes (fresco, linocut, miniature, panorama, spray print, trompe l’oeil, etc.), which are used to further classify artists and artworks. Using these very refined categories (16 in total) and based on the full set of genomes, we trust that the control group we chose is meaningful and fits well the treated group of appropriation artists, beyond the observable characteristics and information on artists and artworks from auction listings that we control for in the later analysis and turn to next.

As a second source, we compile trade data on auction outcomes from Artprice (2020). The Artprice database contains auction records on more than 12 m. artworks from around 6000 houses worldwide and auctions since the 1960s (Artprice, 2020). First, for unique artists from our initial Artsy list, the Artprice database allows us to combine information on approximate production and the supply of works over time, i.e., we can establish the sales catalog of works for appropriation artists, the so-called catalog raisonné. We can match auction data for 1025 appropriation artists (out of the total 1901 artists) and another 1162 similar artists (out of the total 2362 artists). Descriptives for matched and unmatched artists from Artsy samples yield very similar results.^11^ Second, we gather additional information from Artprice webpages on the level of the individual artwork (for example, the work’s title, size, medium, date of creation, price estimates, and ‘hammer prices’) and the level of the auction house (for example, location and auction date). Notably, most auction markets are heavily concentrated around a few superstars which also applies to Appropriation Art. Andy Warhol, Damien Hirst, and Roy Lichtenstein alone account for more than a quarter of all auctions recorded for appropriation (-close) artists.^12^

### Descriptive statistics

Table 1 gives a descriptive overview of the matched sample of appropriation artists compared to similar artists. This dataset contains 171,573 auction results for appropriation artists and 198,073 auction results for artists in the control group, and thus, a total of $n =$ 369,646 outcomes from art auctions recorded over a forty-year period since 1980. The way the data is structured, we can have multiple auction listings per artist in the same year, even when aggregating some of the information (e.g., the number of yearly auctions). As, among other things, we don’t want to lose the more granular information on the level of the artwork and the auction (artwork medium, auction house name, location, etc.), we keep all observations for each artist-auction-year combination in the dataset. Note further that the panel is highly unbalanced, i.e., some artists in our sample may have no observations in a given auction year, while some artists may have multiple auction records on the very same day.

**Fig. 1:**
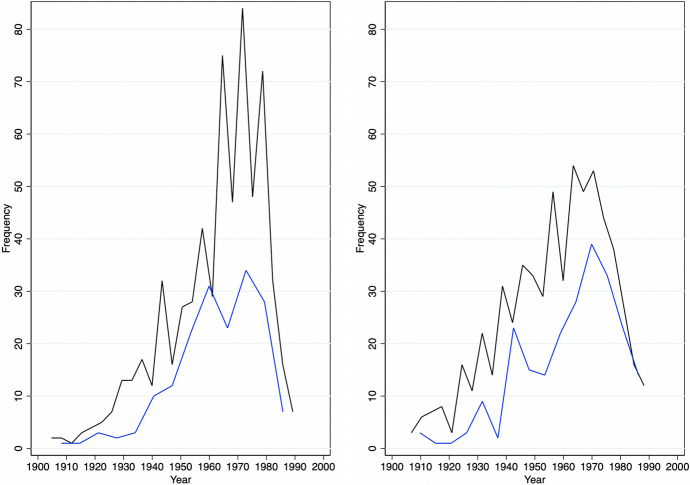
Panel Distribution of Artists’ Year of Birth. *Note* This figure shows the birth year of appropriation artists (left) and similar artists (right) by their gender. The blue lines indicate female artists and the black lines represent male artists’ birth year. The two figures are restricted to artists born in 1900 or later, 26 artists are excluded, (*n* = 2159)

**Fig. 2:**
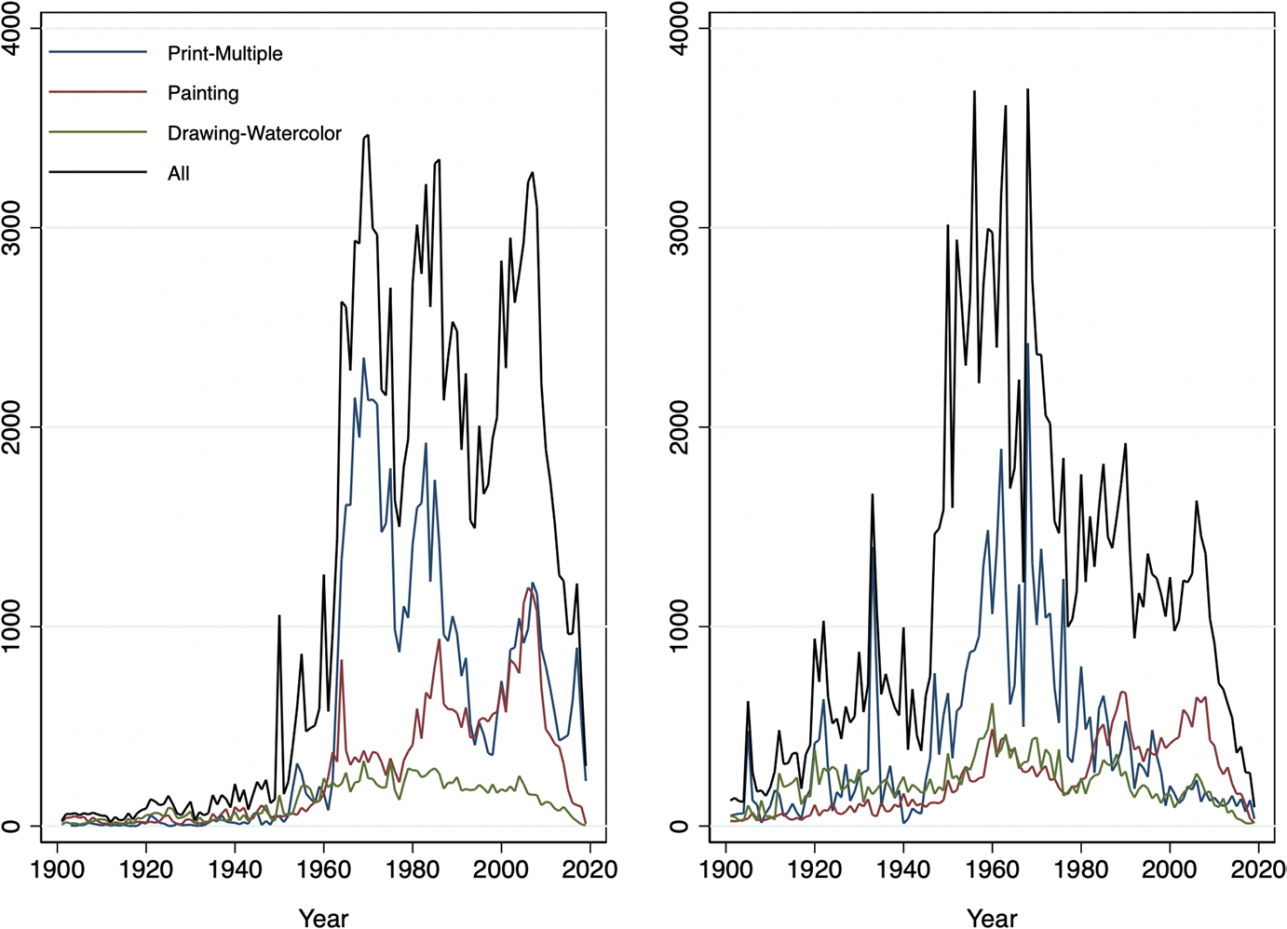
Panel Distribution of Artworks’ Year of Creation and Selected Artwork Medium. *Note* This figure shows the total number of artworks (black line) and corresponding years of creation. The colored lines represent the distribution of selected artwork mediums. The left (right) panel presents appropriation artists (control artists). Samples are restricted to unique artworks (based on titles) and those created after 1900, (*n* = 221′892)

**Fig. 3:**
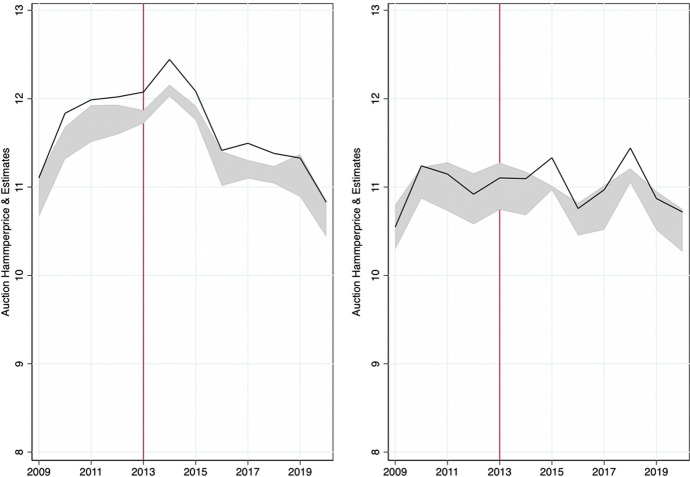
Panel distribution of auctions’ hammer-prices and price estimates ranges. *Note* This shows the yearly log-transformed mean of the price-estimates ranges (gray-shaded area) and realized auction hammer prices (black line). The left (right) panel shows auction outcomes for appropriation (control group) artists

On the one hand, appropriation artists were, on average, born in 1962 (Fig. 1) and female artists account for roughly one-fifth of the total sample. The average artwork was created in 1984 (Fig. 2). And, the data show interesting cyclical and, if anything, weakly increasing trends in the production and supply of new works as recorded in the auctions data. This is in line with our expectations as Appropriation Art is a relatively recent art movement. Furthermore, in three-quarters of total auctions by appropriation artists lots were sold and traded at a mean (median) hammer price of 125’590 (5’000) USD (Table 1, Fig. 3). Figure 2 shows that most of these artworks (medium) were multiple-prints (46%) or paintings (22%).^13^

**Table 1:** Descriptive statistics: overall distribution

	Mean	SD	Min	Max	OBS
*Appropriation artists*					
Artists-level					
Birthday	1962.35	17.21	1879	2010	840
Deathday	1998.34	18.56	1927	2020	79
Female	0.22	0.42	0	1	876
G mixed media	0.35	0.48	0	1	1025
G contemp. conceptualism	0.24	0.42	0	1	1025
G eng. with mass media	0.20	0.40	0	1	1025
G layered images	0.08	0.28	0	1	1025
G neo-conceptualism	0.01	0.11	0	1	1025
G photographic source	0.13	0.33	0	1	1025
G pict. generation	0.01	0.12	0	1	1025
G use of vintage img.	0.05	0.22	0	1	1025
Auction-level					
Artwork creationyear	1984.71	19.51	1854	2019	141,527
Artwork size m2	0.71	1.13	0	53.3	145,476
Artwork size m3	0.45	2.88	0	174.96	13,416
Auction year	2011.1	6.94	1983	2020	171,573
Auction estimate (low) USD	86,745.83	1,068,082	0	1.27e+08	170,248
Auction estimate (up) USD	115,903.5	1,028,728	0	7.00e+07	145,924
Auction hammerprice USD	125,590.3	1,369,431	−3	1.27e+08	121,471
Lot not sold	0.26	0.44	0	1	171,573
Number of auctions**	434.48	632.279	1	2230	171,573
% of U.S. auctions*	0.289	0.261	0	1	171,573
*Control artists*					
Artists-level					
Birthday	1956.22	23.63	1875	1990	895
Deathday	1992.27	32.64	1771	2019	130
Female	0.27	0.44	0	1	960
Auction-level					
Artwork creationyear	1966.9	25.08	1869	2020	143,256
Artwork size m2	0.47	1.23	0	309.68	161,619
Artwork size m3	0.35	1.56	0	78.40	10,316
Auction year	2010.63	7.61	1983	2020	198,071
Auction estimate (low) USD	37,328.79	713,345.5	0	1.40e+08	196,086
Auction estimate (up) USD	56,311.14	692,166.7	0	5.91e+07	160,171
Auction hammerprice USD	52,204.96	911,577.4	4	1.60e+08	141,670
Lot not sold	0.255	0.44	0	1	198,073
Number of auctions**	893.34	1512.5	1	4406	198,073
% of U.S. auctions*	0.248	0.268	0	1	198,073

* and ** are computed as the year-level of ‘number of auctions’ and ‘percentage of U.S. auctions,’ and here shown on the artwork level, the panel data are highly unbalanced among dates and artists. (*) The between standard deviation of appropriation (control) artists is 0.38 (0.39) and the within deviation is 0.11 (0.11) for the percentage of U.S. auctions. (**) The between standard deviation of appropriation (control) artists is 67 (113) and the within deviation is 245 (311) for yearly auctions

**Fig. 4:**
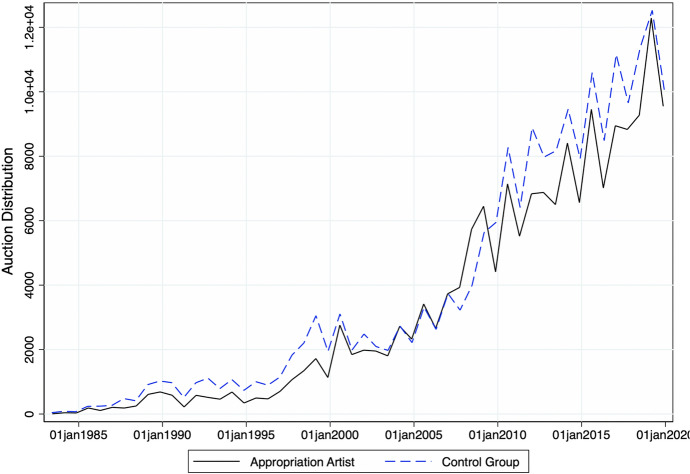
Panel Distribution of Auction Dates. *Note* This figure shows the historic auction distribution (freq) of the total numbers of auctions for appropriation artists (black line) and control artists (blue dotted line), (*n* = 369,646)

On the other hand, similar artists in the control group were born, on average, in 1956, and female artists account for roughly one-fourth of the total (Fig. 1). Figure 4 compares total auctions of artworks by appropriation artists with those recorded for similar artists throughout the observation period. There is an increasing trend in the total number of auctions for each group. This seems to be due to a higher fraction of younger artists entering auction markets as time progresses as well as, eventually, more complete data coverage of auctions over time in the underlying Artprice database. Average (median) hammer prices for similar artists stood at 52,204 (3030) USD (Table 1 and Fig. 3). The difference in average auction prices when compared to appropriation artists is driven by a few outliers and superstars who achieve higher auction results constantly. We, therefore, log-transform the dependent variable ‘hammer price’ in our estimates (for more details, also see Sect. 3.3). Similar to the treatment group, in one out of four auctions for similar artists, lots were not sold.

Moreover, the highest percentage of appropriation artists originate from the U.S. (31%), followed by British (10%), French (5%), and German (5%) artists. In terms of their work location, artists frequently reside in New York (U.S.) (9%), London (U.K.) (6%), L.A. (U.S.) (6%), Berlin (Germany) (4%) or Brooklyn (U.S.) (4%). Geographic origins and locations are comparable for control and appropriation artists and only a few data caveats apply.^14^

Once again, Fig. 4 shows an increasing trend of auction records over time. Most of the auctions take place in auction houses in the U.S. (29%), followed by houses in the U.K., Germany, and France. Moreover, the most prominent auction houses in our sample in terms of the total number of auctions are Christie’s, Sotheby’s, and Phillips (all of UK origin but with satellite houses around the world). The top ten auction houses for similar and appropriation artists are listed in Table 2, accounting for roughly 40% of all records.

**Table 2:** Top 10 auction houses

Appropriation artists		Control artists	
Auction house	Observations	Auction house	Observations
Christie’s	25,510	Christie’s	26,054
Sotheby’s	22,302	Sotheby’s	20,953
Phillips	10,559	Bonhams	7317
Bonhams	4304	Phillips	5016
Artcurial S.V.V.	3676	Swann Galleries	3877
Van Ham Kunstauktionen	2471	Artcurial (S.V.V.)	2934
Cornette De Saint CYR M.	2309	Grisebach	2110
Dorotheum	2005	Lempertz 1792	1789
Lempertz	1962	Mainichi Auction Inc.	1757
Swann Galleries	1962	Dorotheum	1755
1471 other houses	94,489	1864 other houses	124,511

This table shows the distribution of the top 10 auction houses (any country) by appropriation (left) and similar artists (right). The last line gives total auctions/observations by all other houses

**Fig. 5:**
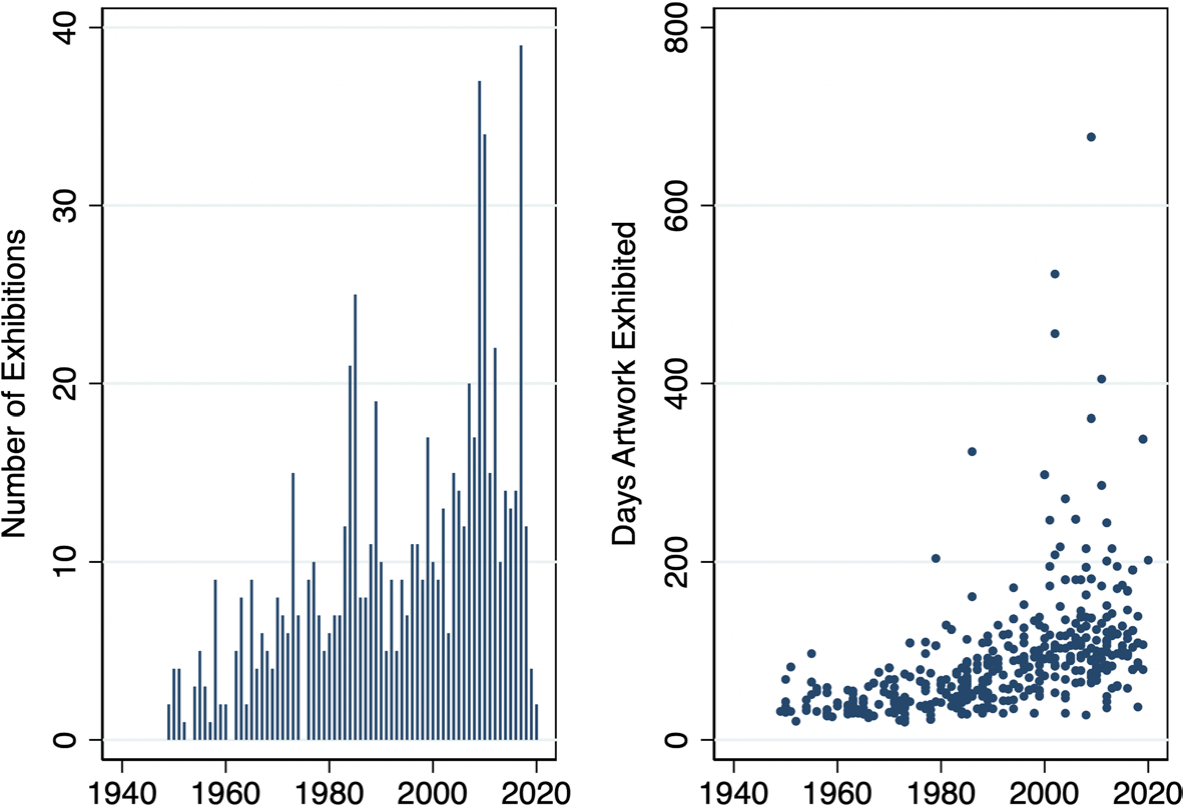
MET appropriation artwork exhibitions. *Note* This figure shows the total number of exhibitions (left) and the total number of days an artwork was exhibited (right) of Appropriation Art artworks (based on a top-100 subsample of artists) in the Metropolitan Museum of Modern Art (MET) New York, (*n* = 521)

By exploring museum collection and exhibition data around Appropriation Art and appropriation-close artworks, we provide further descriptive evidence on the public interest and continued market relevance of the Appropriation Arts following the 2013 decision. For this purpose, we create a subsample of the top-500 appropriation artists (in terms of Artsy ranking) and manually link them to the Metropolitan Museum of Modern Art New York (MET) collection database.^15^ On the left-hand side, Fig. 5 shows the total yearly number of exhibitions of appropriation artworks in the MET Museum or artworks on loan to other museums. Overall, it shows an overall increasing trend. On the right-hand side, when taking a look at the total number of days these artworks were exhibited, trends are as well increasing but they seem slightly less pronounced for more recent years. In general, appropriation artworks are exhibited more often, but also for longer periods. In addition, based on the vast MET collection as one of the most important contemporary art collections in the U.S. (if not the world), we find further support for the importance and continued interest in the Appropriation Arts in recent years as more than 10% of our Appropriation Art subsample of artists were found in the MET collection. Based on this descriptive evidence, the indirect liability of intermediaries such as museums^16^ and arguably greater legal uncertainty in the copyright system does not seem to have systematically limited the availability and dissemination of possibly infringing artworks in these (public) spaces. Whether this result would continue to hold for museums in a multivariate setting or a more detailed, artwork-level analysis is an interesting endeavor we leave for future research.

### Empirical framework

We begin the empirical section with a historic auction price analysis applying hedonic price models to artworks on sale. Auction prices and market mechanisms are central to better understanding the incentives to create and the valuation of artworks and can help explain the cost of creating and distributing works of art (Ashenfelter & Graddy, 2003). Thus, based on a burgeoning number of empirical studies on art auctions (Ashenfelter & Graddy, 2003, 2006, 2011; Beggs & Graddy, 2009; Mei & Moses, 2001), we construct prices indices for our group of artists (appropriating artists and control artists), analyzing fixed components of the price functions as hedonic characteristics, and deploying the following type of multiple high-dimension fixed-effects regression models (Correia, 2019):

$$\begin{aligned} p_\textrm{itc} = \alpha + \varvec{X'}_\textrm{itc} + \phi _I + \rho _Y + \mu _C + \epsilon _\textrm{itc} \end{aligned}$$

where $p_\textrm{itc}$ is the log-transformed auction hammer price, $\phi$ represents artist fixed-effects, $\rho$ auction year fixed-effects and $\mu$ country fixed-effects. Finally, we capture in $\varvec{X'}$ several artwork-specific characteristics of interest, such as the artwork age at auction, artwork size, or artwork medium. We run separate regressions for the appropriation artists and the control group.

**Fig. 6:**
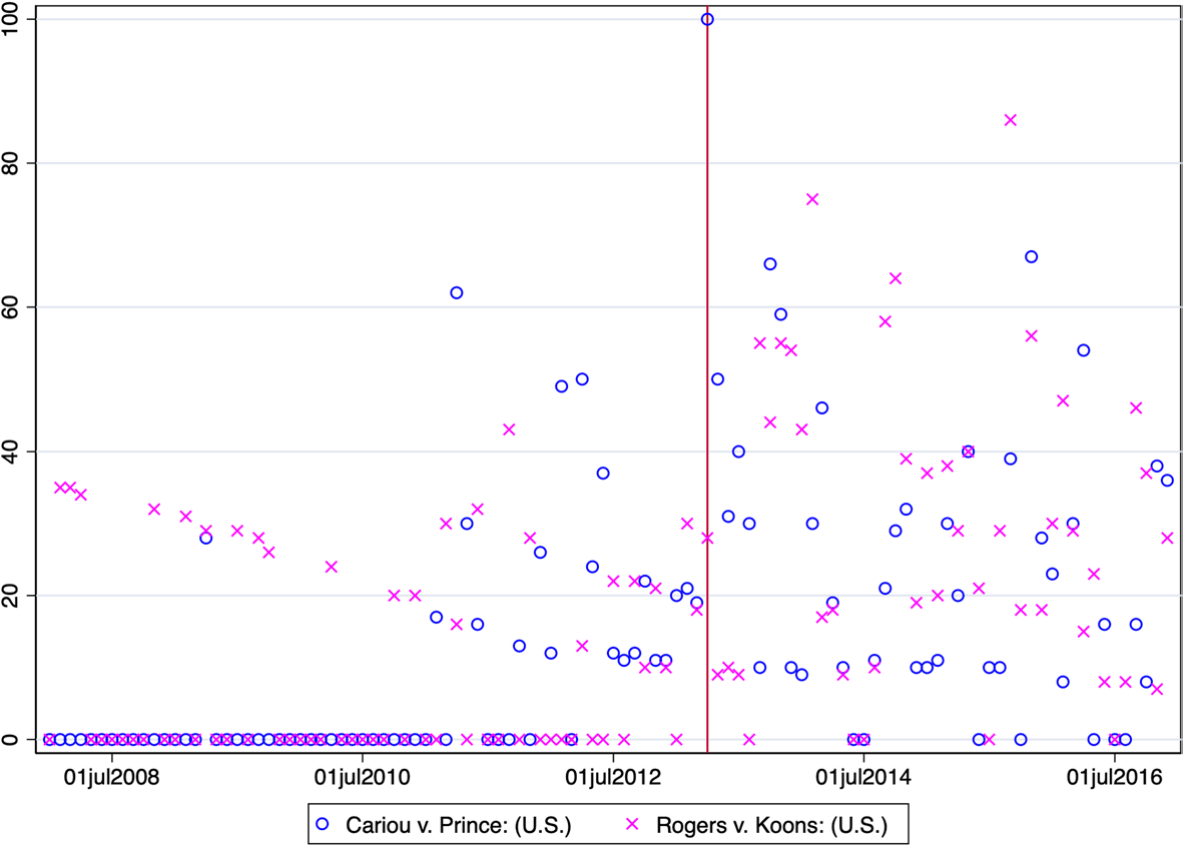
Google Search Volume: Cariou v. Prince. *Note* This figure shows online searches in the United States between 2008 and 2016 for two sets of keywords: ‘Cariou v. Prince’ (blue circles) and relative to ‘Rogers v. Koons’ (pink crosses) around the 2013 court decision (red vertical line). Google search data are normalized

In the next step, our empirical strategy exploits the 2013 court decision on intermediary liability as an exogenous institutional shock using a differences-in-differences design (see also Sect. 2.2 on ‘Copyright Rules and the Reuse of Images’ where we discuss the importance of the court decision from an economic and legal point of view in greater detail), using the important hedonic characteristics of artworks as control variables. We, therefore, tighten our data sample for estimating our main results closely around the higher court decision with auction years dating from 2010 to 2020 to analyze short-mid-term secondary market reactions. In additional robustness checks, we estimate the main results based on 2007–2020 and longer time-frames. At its core, while the decision may have changed what appropriation ‘practices’ were considered exempt from litigation, it left the relevant liability question open and pending, increasing the perceived litigation risk for traders of artworks, and we are specifically interested in post-2013 changes. Based on Google trends data, Fig. 6 shows online searches for sets of google keywords, ‘Cariou v. Prince’ in the left panel (i.e., common ways to search for legal cases in the U.S.). This gives rise to two interesting takeaways. First, in line with our empirical strategy, around the 2nd Circuit decision cutoff date in April 2013, we see a systematic and large spike in searches on Google. Second, we can compare search traffic around the decision to searches around the ‘Rogers v. Koons’ case, another prominent court decision (1992) that is frequently discussed by legal scholars in the context of Appropriation Art and fair use.^17^

The identification strategy exploits two alternative differences-in-differences approaches to estimate the effect of the prominent *2013 Cariou v. Prince* court decision, a change in copyright jurisprudence, and on intermediary liability, so to speak Adler (2016). A differences-in-differences analysis is a common research design to estimate the causal effects of such a change in law practice or policy change [for an application to changes in copyright laws see, for example, Kretschmer and Peukert (2014); Watson (2017b)]. A first strategy considers the global auction market for Appropriation Art as potentially treated^18^ in the post-2013 period, and thus we control for auctions and artworks by similar artists that we can identify based on the Artsy genome information. A second strategy compares only auctions for appropriation artworks in the U.S. (treated group) to auctions of the same art movement outside the U.S. (control group). We assume that artists themselves do not directly respond to a particular court decision in a copyright case by shifting ‘appropriation (art) practices,’ for example, by increasingly reusing non-infringing materials from the public domain in new artworks. This can be explained by the fact that, for most Appropriation Art artists, knowingly appropriating (copyrighted) objects and images is at the very center of their artistic strategy and practices. A recent report on the visual arts sector in the U.S. Aufderheide et al. (2014) states that ‘the artists we interviewed typically did not want to think about copyright as they made their work for fear of it interfering with their creativity,’ and ‘artists whose work had a political or social-commentary cast also were emphatic that typically unlicensed quotation from copyrighted work was a core part of artistic practice.’ This is anecdotal evidence that changes in core practices are very unlikely to occur. Therefore, we expect artists to be faithful to the original ‘conception’ of the Appropriation Art (movement). Most importantly, however, the majority of artworks in our samples were created a long time before the court verdict. The mean year of creation for artworks by appropriation (similar) artists is 1984 (1966), respectively. So, we would expect the impact on changing practices, if any, to be negligible and limited to a few, more recently created artworks. Given the various copyright litigation cases against artists *and* the prominent contributory liability ruling in 2013, we expect responses to a change in the (visual art) fair use jurisprudence on *secondary market trade* of appropriation artworks.

An important task is to construct meaningful and precise dependent variables that will capture and reflect potential changes of (intermediary) liability around artworks. Here, we are interested in the short- or medium-term reactions on secondary art markets. In the first empirical setting, we make use of the fact that not all auctions result in a sales success, and hence, the auction houses will either report the hammer price or that the artwork has not been sold, i.e., ‘lot not sold.’ This is the case for roughly 30% of all auction records, in both our treatment and control samples. We thus run logit models for the probability that an appropriation artwork is a sales success when compared to similar artists and their auction successes before and after the event in April 2013. Arguably, a focus on immediate sales success captures the short-term effect of the policy change. If auction schedules only can be adjusted slowly to the changes in the legal environment and the supply of artworks is to a certain degree ‘sticky’^19^, we might well observe immediate demand responses via the observed changes in sales probabilities. The reactions on the demand side might well reflect general changes in perceived risks around artists and market values, also from a financial investors’ point of view. Generally, with an increase in risks, costs to curate and offer artworks might increase. For instance, auction houses might have to engage costly lawyers, or compensate other third parties involved in (potential) lawsuits. In *Cariou v. Prince*, initially, also the RCS MediaGroup which printed the exhibit catalog was sued for copyright infringement. As a consequence, auction houses might charge potentially treated artworks at higher commissions, and buyers are not willing to meet the desired auction reserve price anymore. Demand-side reactions therefore might capture insightful secondary market reactions, as we argue in our paper. In the medium term, however, auction houses and galleries will adjust their supply to the changes in potential litigation risks of hosting and selling appropriation artists, and these artworks might no longer (or less often) appear on the secondary market. In turn, another set of regressions focuses on the total number of auctions. Arguably, this captures the medium-term effects on the total trade of appropriating artworks which potentially include copyright-infringing materials before and after the decision.

Finally, regressions take a closer look at the U.S. market outcomes. Here, we focus on the percentage of artworks by an appropriation artist that is hosted in U.S. auctions (relative to non-U.S. auctions) compared to the percentage for a similar artist and her works in the pre- and post-2013 periods. This effect might well capture whether or not appropriation artworks increasingly shifted to auction houses outside the U.S. and other jurisdictions, and whether the changes in perceived litigation risks among secondary market intermediaries in the U.S. led to a ‘relocation’ of global trade in due course. To set up our baseline model more formally, let

1 $$\begin{aligned} Y_\textrm{itc}^k & = \alpha + \beta _1 \textrm{Post}_t + \beta _2 \textrm{Treat}_i +\delta (\textrm{Treat}_i \times \textrm{Post}_t) \nonumber \\ & \quad+ {\varvec{X}} + \mu _i + \phi _t + \gamma _c+ \epsilon _\textrm{itc}, \end{aligned}$$

where $Y_\textrm{it}^k$ are the outcome variables (*k*) for auction results of artist *i* at date *t*. $\textrm{Treat}_i$ identifies the group of treated appropriation artists and $\textrm{Post}_t$ all auctions scheduled after April 2013. The differences-in-differences coefficient of interest is captured by $\delta$. We include year fixed effects $\phi _t$, country fixed-effects $\gamma _c$, artist fixed effects $\mu _i$, and a vector of hedonic artwork controls *X* (elicited from the hedonic art auction price regressions from the initial analysis) to the baseline model.

A second, alternative identification strategy limits the data to appropriation artists only and compares their U.S. (treated) to non-U.S. auctions (control group) before and after the prominent court decision. In this way, the strategy also addresses potential boundary and composition issues for treatment and control groups. Arguably, not all artists in the treatment group are incorporating copyright-protected material in each of their artworks, and the potential infringement of the derivative or the resulting work can only be considered an approximation. In a similar vein, some artworks and art practices by similar artists might involve the reuse of already existing, original works created by other artists. This will make it harder to establish clear-cut boundaries between treatment and control groups.

Baseline models include artists- and year-fixed effects (FE) and, clustered standard errors at different levels or bootstrapped standard errors, taking into account the plausible auto-correlation in the data as discussed in Bertrand et al. (2004) for differences-in-differences estimations. The FE panel regressions allow us to observe the coefficient of interest, $\textrm{treat} \times \textrm{post}$, and we control for unobserved artist-specific and time-invariant heterogeneity as well as time trends in the auctions data with year FE. However, as being an appropriation artist (or not) is not a time-variant characteristic of the way our initial data is structured, we cannot separately report effects for our treated group coefficient appropriation. Furthermore, next to the baseline models, we include artwork-level specific controls (hedonic characteristics, such as artwork size, artwork medium, and artwork age at auction date) or auction-house-specific controls (top auction houses^20^ and sales country), or both in alternative model specifications. Furthermore, fixed-effects and differences-in-differences estimators are based on the idea of time- or group-invariant omitted variables (Angrist & Pischke, 2008), and potential endogeneity could lead to incorrect inferences. Thus, unobserved time-variant heterogeneity in the group of treated artists might still be correlated with the error term. Given the quasi-experimental framework, our large-scale auction dataset, and various control variables, we assume that the potential omitted variable bias is less of a concern, and careful causal inference is appropriate.

### Limitations

The overall data and empirical approach are not without limits. First, it is a difficult task to identify the group of treated artists. On the one hand, appropriation artists are not a heterogeneous group to identify, we are only able to approximate this group via genome records on the artist level publicly available in the Artsy data. I.e., unfortunately, we cannot explore the ‘appropriation level’ of individual artworks. On the other hand, artists and market intermediaries such as auction houses could perceive and deal with the potential litigation risks differently. Some agents have different risk attitudes at the outset and will respond more than others to changes in copyright frameworks. Accordingly, changes in their supply of these artworks might be more pronounced too.

Furthermore, based on the data, we are not able to identify the underlying original work that has been appropriated and fully establish the associated copyright status (and, accordingly, whether or not rights have been cleared). Such a strategy would require artwork-level metadata and licensing records which are, unfortunately, not available to us. Survey evidence for practicing visual artists and nonartist professionals (art historians, museums, etc.) in the U.S. visual arts sector suggests, however, that this is of limited concern for the analysis Aufderheide et al. (2014). Practicing artists are much less likely to pay copyright fees than nonartist professionals. They were reported to rarely or never (82% for artists vis-á-vis 40% for nonartist professionals) or frequently/occasionally (17% for artists vis-á-vis 60% for nonartist professionals) clear rights and pay access fees to rightsholders before incorporating existing works Aufderheide et al. (2014). In addition, technological (digital) change may also affect reuse practices over time as it potentially impacts access and production costs when artists are appropriating existing materials. We seek to address some of these issues in the analysis.

Finally, monitoring of trade in secondary markets is limited to auction data as we do not have access to private sales data or sales on primary markets such as galleries, some of which are confidential or not collected on a systematic basis. Still, we trust that the auction data provide us with a representative, multi-year view of global markets for the Appropriation Art and artworks on sale by similar artists.

## Results

### Hedonic price models

In this section, we employ a standard hedonic pricing approach to art auctions for both groups of artists. As shown in Fig. 3 and as discussed in the result section below, at large, auction prices do not systematically differ between the two groups. However, in this series of hedonic price models, we more comprehensively assess price formation for artworks in both groups, by saturating models with country, year, and artist fixed-effects to better understand auction results and their relation to artwork characteristics. We regress on the historic auction price outcomes for auctions held over the entire period between 1983 and 2020.

We present the hedonic price estimations in Table 3 where the dependent variable is the log price of an artwork realized at an auction (i.e., ‘hammer-price’). Model (1) includes artist, and year fixed-effects, model (2) adds country fixed-effects, and model (3) additionally controls for all auction houses in the data sample of appropriation artists, the same model structure applies for the control group of artists shown in model (4–6). Some insightful and reasonable differences in price formations can be noted. While outcomes for appropriation artworks provide for significant large positive coefficients of ‘audiovisual-multimedia’ artworks, outcomes for control artists show the same direction of effects, effects render insignificant. The opposite holds for artworks created with the medium ‘lightings,’ where appropriation artists show negative effects, while this shows a positive sign in the control group outcomes. This, however, can be explained by the relatively small number of observations for this artwork medium type. Furthermore, it seems that artworks by appropriation artists achieve negative correlations of prices with the artwork medium ‘objects,’ while for the control artists sample, we find a positive, however, insignificant coefficient.

Notably, appropriation artworks and control artworks share many common price characteristics as revealed in Table 3. The $artwork-size$ and the $artwork-age$ (at the auction) both yield positive effects on auction hammer-prices, in line with, e.g., results in (Beggs & Graddy, 2009). The same goes for different kinds of mediums. On the one hand, classical artwork mediums such as *paintings* (at the 0.001%-level) and $sculpture-volume$ are both positively correlated with log-transformed prices. On the other hand, if several copies of an artwork exist, i.e., $print-multiple$, prices are negatively correlated for both groups.

Finally, auction outcomes for artworks traded at major auctioneers, for example, at Sotheby’s, Christie’s, Phillips, and Bonhams, are all highly positive and significantly correlated with the hammer-price outcomes, again in line with recent work on hedonic pricing (Ashenfelter & Graddy, 2003; Beggs & Graddy, 2009). Although in models (3) and (6), we control for *all* auction houses with fixed-effects, results suggest that they do not systematically differ where we capture the most important auction houses only and do not distinguish auctioneers (models 1, 2, 4 and 5).

Overall, models appear to be adequately specified and most controls yield estimates very much in line with the existing literature. This provides for insightful price analysis and helps us to better understand the auction results of both groups of artists. Indeed, this is not unexpected, as some of the literature on auction prices [for an overview, see, for example, Ashenfelter and Graddy (2003)] suggest that the heterogeneity of the auction price indices is best addressed by hedonic price models on the level of the individual artwork.^21^ That is to say, the price composition of artworks is somewhat complex to analyze, and so we can expect to observe no direct price responses for traded artworks after the policy change as the effects cannot be studied based on a repeat sales panel for the very same artwork. In this way, data structures, ultimately, constrain our ability to further inspect price effects.

**Table 3:** Hedonic price models

DV: log(auction hammer price)	(1)	(2)	(3)	(4)	(5)	(6)
	Appropriation artists			Control artists		
(Artwork size)^2^	0.273***	0.269***	0.266***	0.321***	0.315***	0.308***
	(6.92)	(6.94)	(7.21)	(8.74)	(8.70)	(8.41)
Audiovisual-multimedia	1.996***	1.893***	1.680***	0.938	0.770	0.989
	(4.54)	(4.46)	(4.18)	(1.26)	(1.03)	(1.35)
Ceramic	−0.278	−0.260	−0.290	−0.748	−0.785	−0.858*
	(−0.79)	(−0.72)	(−0.84)	(−1.74)	(−1.86)	(−2.12)
Drawing-Watercolor	0.461*	0.468*	0.312	0.515	0.505	0.396
	(2.31)	(2.25)	(1.53)	(1.11)	(1.10)	(0.89)
Furniture	0.148	0.134	0.189	1.546*	1.540**	1.469**
	(0.95)	(1.40)	(1.67)	(2.56)	(2.58)	(2.61)
Lightings	−0.487***	−0.453***	−0.443***	1.119	1.154*	1.058*
	(−3.59)	(−4.67)	(−5.00)	(1.95)	(2.05)	(2.00)
Objects	−0.946***	−0.932***	−0.881***	0.830	0.797	0.642
	(−4.95)	(−4.03)	(−4.16)	(1.73)	(1.70)	(1.45)
Painting	1.724***	1.716***	1.561***	1.761***	1.752***	1.625***
	(6.85)	(6.79)	(6.49)	(3.73)	(3.80)	(3.61)
Photography	−0.192	−0.184	−0.302	−0.199	−0.203	−0.249
	(−0.90)	(−0.83)	(−1.30)	(−0.43)	(−0.44)	(−0.57)
Print-multiple	−0.894**	−0.875**	−0.900***	−1.258**	−1.267**	−1.214**
	(−3.10)	(−3.16)	(−3.67)	(−2.94)	(−3.01)	(−3.01)
Sculpture-volume	1.085***	1.091***	0.938***	1.037*	1.018*	0.920*
	(4.92)	(4.89)	(3.88)	(2.46)	(2.46)	(2.35)
Tapestry	−1.036**	−1.018**	−1.008**	−2.114***	−2.013***	−1.998***
	(−3.07)	(−3.14)	(−3.10)	(−4.55)	(−4.38)	(−4.47)
Artwork age at auction	0.00109	0.00132	0.00128	0.00951**	0.00928**	0.00764**
	(0.38)	(0.46)	(0.46)	(2.83)	(3.02)	(3.07)
Phillips	1.093***	1.070***		0.969***	0.952***	
	(17.24)	(14.16)		(12.17)	(9.98)	
Sothebys	1.356***	1.362***		1.242***	1.250***	
	(19.89)	(17.48)		(13.57)	(11.96)	
Christies	1.153***	1.158***		1.027***	1.046***	
	(16.45)	(15.17)		(10.63)	(10.21)	
Bonhams	0.496***	0.475***		0.362***	0.379***	
	(4.73)	(5.92)		(5.73)	(4.68)	
Year FE	$\surd$	$\surd$	$\surd$	$\surd$	$\surd$	$\surd$
Artist FE	$\surd$	$\surd$	$\surd$	$\surd$	$\surd$	$\surd$
Country FE	No	$\surd$	$\surd$	No	$\surd$	$\surd$
Auction House FE	No	No	$\surd$	No	No	$\surd$
Cluster SE	Artist	Artist	Artist	Artist	Artist	Artist
*N*	95533	95529	95332	89822	89819	89550
${R}^2$	0.651	0.659	0.696	0.624	0.633	0.684

This table shows the historic analysis (1983–2020) of price formations of artworks by appropriation artists and control artists, as specified in chapter 3.3

*t* statistics in parentheses

*$$p<0.05$$, **$$p<0.01$$, ***$$p<0.001$$

### Intermediary liability and secondary market effects

As described in Sect. 3.3, we estimate the causal impact of the higher court decision on liability of intermediaries on trade and availability of artworks by appropriation artists (treatment group) relative to a group of control artists and their works. As an exogenous shock, this decision changed the perceived litigation risk for market intermediaries.

We first turn to differences-in-differences models. For that purpose, we tighten the time frame with auction years 2010–2020 closely around the date of interest in 2013, as our DiD strategy tries to identify the short- to mid-term effects of a change in copyright practice on secondary market effects. Table 4 provides summary statistics for our final estimation sample, showing sample characteristics before and after the court decision and samples by observational group (appropriation vs. similar artists).

**Table 4:** Descriptive statistics: pre/post-samples

Variable	Appropriation artist				Control group			
	2010–2012 (pre)		2013–2020 (post)		2010–2012 (pre)		2013–2020 (post)	
	Mean	SD	Mean	SD	Mean	SD	Mean	SD
Birthyear*	1955.94	18.28	1959.05	18.54	1947.20	23.25	1950.36	22.93
Deathyear*	1996.59	19.78	1996.65	19.63	1994.19	21.17	1994.02	22.06
Female*	0.15	0.36	0.16	0.37	0.17	0.38	0.19	0.39
Artwork age at auction	25.83	17.94	27.39	19.34	44.65	24.36	47.49	25.43
Artwork sreationyear	1985.37	17.95	1989.06	19.44	1966.59	24.36	1968.97	25.47
Artwork size m^2^	0.74	1.12	0.67	1.09	0.44	0.89	0.46	1.52
Artwork size m^3^	0.50	2.17	0.39	2.29	0.41	1.97	0.30	1.49
Auction year	2011.20	0.95	2016.48	2.00	2011.23	0.95	2016.45	2.00
Philipps	0.08	0.27	0.06	0.24	0.03	0.16	0.02	0.15
Christies	0.15	0.36	0.10	0.30	0.15	0.35	0.09	0.29
Sothebys	0.11	0.31	0.08	0.28	0.08	0.26	0.08	0.27
Superstar	0.40	0.49	0.42	0.49	0.45	0.49	0.38	0.49
Log (Number auctions)**	3.72	1.38	3.78	1.46	3.61	1.42	3.83	1.5
US auctions	0.28	0.24	0.26	0.24	0.22	0.23	0.25	0.25
Hammerprice (log)	8.79	2.15	8.38	2.21	8.17	1.91	8.04	1.97
Lot not sold	0.30	0.46	0.30	0.46	0.29	0.46	0.27	0.45
Obs max.	29,551		84,610		35,513		96,930	

Reported data are on artist-artwork-level and are based on a highly unbalanced panel of artists. The reported year-panel is based on estimates derived from baseline models. The post-periods include auction dates after the U.S. court ruling of interest in April 2013

*Averages reported based on the artist panel

**Superstars excluded and a dummy separately reported in this table

Table 5 presents baseline estimates for the dependent variable ‘number of auctions.’ For sake of clarity, the table only reports the coefficient of interest, and the interaction of the treatment group with the post-2013 period dummy. Models report multi-way fixed-effects (artist, auction-year, and country fixed-effects) and the panel/observation period is restricted to the years 2010–2020 inclusive (with the exception of model 6, using the overall panel period).^22^ We estimate a robust negative effect of minus 63 to 67 auctions for our group of treated artists following years after the U.S. court decision. We further note that using our preferred model specification (artist-fixed effects and clustered standard errors at the artist level) estimates yield no significant effect. Clustering standard errors has a large impact on the significance, as we demonstrate in models 2 (on artwork-creation-year level) and 3 (on country level). We therefore carefully interpret this coefficient as weak evidence for a negative sign and direction of the effect. Although our preferred models are calculated with artist-fixed effects, we can rule out potential bias on baseline results from so-called ‘superstar’ artists experiencing a significantly higher number of yearly auctions, as descriptive statistics illustrate. We expect this to be an important influencing factor for our estimates and the total ‘number of auctions’ we observe. This is because, for various reasons, the underlying economic mechanism also partially builds on the popularity and prominence of the artist at stake.^23^

Accordingly, models 4 to 6 re-run and present model specifications, now using an outlier robust log-transformed dependent variable of artists’ yearly number of auctions. We construct a superstar dummy and introduce in model (4) and (6) a triple-interaction $treat \times post \times superstar$ (not reported). The coefficient of interest, $treat \times post$, stays negative even in this model specification (with a t-statistics of 1.95 in model 4).^24^ Furthermore, model (5) is computed based on a smaller sample, excluding superstars from the estimation sample. Also here, the coefficient of interest stays robust and yields the expected negative coefficient. Moreover, once we widen the time frame to include all auctions between 1983 and 2020, including all fixed-effects levels, and the triple-superstar interaction term, the coefficient continues to be negative, however, on a smaller magnitude of minus 0.0057 (model 6).

**Table 5:** Baseline results: number of auctions

	(1)	(2)	(3)	(4)	(5)	(6)
	MWFE			DV: log(*N* auctions)		
Treat $\times$ post	−62.78	−66.58***	−66.58***	−0.0866	−0.0844	−0.00573
	(−0.61)	(−4.06)	(−5.90)	(−1.95)	(−1.91)	(−0.09)
Year FE	No	$\surd$	$\surd$	$\surd$	$\surd$	$\surd$
Country FE	No	$\surd$	$\surd$	$\surd$	$\surd$	$\surd$
Artist FE	$\surd$	$\surd$	$\surd$	$\surd$	$\surd$	$\surd$
Cluster SE	Artist	CreationY	Country	Artist	Artist	Artist
Timeframe	2010–2020	2010–2020	2010–2020	2010–2020	2010–2020	1983–2020
*N*	168,544	168,543	168,543	168,543	102,110	257,161
${R}^2$	0.971	0.977	0.977	0.976	0.927	0.958

*t* statistics in parentheses

*$$p<0.05$$, **$$p<0.01$$, ***$$p<0.001$$

This table shows the regression results for the dependent variable ‘number of auctions’ model 1–3 and log(number of auctions) in model 4–6. All results are calculated as specified above. Model 5 using non-superstars, and model (4) and (6) a superstar $\times$ treat $\times$ post triple-interaction (not reported). All models include artwork-specific controls (age at auction, size, medium) and auction-house-specific controls (Phillips, Sotheby’s, Christie’s, Bonhams)

Table 6 reports logit and OLS regression results for the dependent variable ‘lot not sold,’ i.e., a dummy variable for items listed but not sold at auctions. This dummy is 1 if the auction results in a sales success, 0 if the lot is not sold. We, therefore, re-run our baseline results using an OLS regression model (1). As outlined in the empirical framework, the dependent variable should reflect short-term responses on the demand side, and we, therefore, are interested in the observation period to +/− 2 years around the court intervention (i.e., 2011–2015). In this way, we can rule out again possible bias in the estimates from the financial crisis. However, model (7) provides for a comparison using the longer time window and shows that the overall direction of effects does not change. Moreover, arguably, the composition of artworks included in auction samples could potentially change as a response to the court decision. In turn, this could also impact estimated sales probabilities around auctioned and curated artworks. To address this potential selection issue, we provide further descriptive evidence on pre/post-observable characteristics of artworks. Descriptive statistics for each sample shown in Table 4 indicate that, at large, sample composition does not change.^25^ Ultimately, approximately three-quarters of the total artists in the estimation sample contribute to the identification of effects in our unbalanced panel, as they see auctions and are present in both, pre- and post-sample periods.

In all models of Table 6 addressing short-term effects, the coefficient of interest is statistically significant at the 5%-level. This can be interpreted as follows: Artworks by treated appropriation artists have a higher probability of not selling in auctions run after 2013 as compared to similar artists and their artworks on auction sale in the same period.

Models (2 and 3) show regression estimates using a logit model, and an estimated coefficient of around 0.13. Results are robust to the inclusion of year- and country-fixed-effects as model (3) illustrates. When using multi-way-fixed-effects in models (4) to (7), our results continue to hold. The same applies when standard errors are clustered at artist- or artwork-creation-date levels (model 6). Further conditioning models on price estimates do not change results (estimates not reported). Given that, on average, one-fourth of the listed auctions in the treated and control samples do not result in sales success, we discuss the economic significance of our findings in the next paragraph.

At large, even smaller changes in sales success, as well as the changes in the total number of auctions, may have a significant effect on the overall value traded on art markets. Here, we are assuming that artworks did not find alternative sales channels other than the auctions we observe. Based on the ATTs and the estimated changes in the total number of auctions, we can provide a simple back-of-the-envelope calculation: Given that the median (appropriation) artwork sold after 2010 was auctioned for 4500 US-Dollars and the treated group includes 1025 unique artists, the plausible (annual) market value forgone due to artworks not being auctioned in this particular art movement or field is roughly 290–304 m. US-Dollars on a global level. However, this includes the possibility that some of the value forgone was invested in alternative art movements and went to other artwork auctions. Still, to put these numbers into context, auction sales for appropriation and control artists in our data amounted to a total of 2.36 b. US-Dollars in 2014. Moreover, the total sales volume from all public fine art auctions in the same year reached an estimated 24.2 b. US-Dollars globally, according to the Art Market Report 2020 (McAndrew, 2019).

**Table 6:** Baseline results: lot not sold

DV: lot not sold	(1)	(2)	(3)	(4)	(5)	(6)	(7)
	OLS	Logit		MWFE			Timeframe
Treat $\times$ post	0.0265*	0.130*	0.111*	0.0219*	0.0176*	0.0176*	0.00634
	(2.57)	(2.53)	(2.25)	(2.24)	(2.08)	(2.27)	(0.64)
Year FE	No	No	$\surd$	$\surd$	$\surd$	$\surd$	$\surd$
Country FE	No	No	$\surd$	$\surd$	$\surd$	$\surd$	$\surd$
Artist FE	No	No	No	No	$\surd$	$\surd$	$\surd$
Cluster SE	Artist	Artist	Artist	Artist	Artist	CreationY	Artist
Timeframe	2011–2015	2011–2015	2011–2015	2011–2015	2011–2015	2011–2015	1983–2020
*N*	75,247	75,244	75,166	75,245	74,958	74,958	257,161
${R}^2$	0.0256	0.0226	0.0368	0.0433	0.0945	0.0945	0.0901

*t* statistics in parentheses

*$$p<0.05$$, **$$p<0.01$$, ***$$p<0.001$$

Note: This table shows the regression results for the dependent variable ‘lot not sold.’ All models include artwork-specific controls (age at auction, size, medium) and auction-house-specific controls (Phillips, Sotheby’s, Christies, Bonhams)

### Relocation of trade

To reiterate, the main purpose of this paper is to analyze whether market intermediaries responded to the higher court decision on vicarious liability or not. Compared to previous results on worldwide auction trade volumes of treated artists, this section takes a closer look at a potential U.S. auction market shift to non-U.S. jurisdictions due to the prominent court decision in the U.S.

More specifically, we deploy the relative share of U.S. located auctions (in total auctions by the same artists) as the alternative outcome measure. Table 7 presents these baseline estimates. We observe negative and significant effects of around minus 0.0306 to minus 0.0385 in any model specification. When further adding country-fixed effects (2), and year fixed-effects (3) in the multi-way fixed-effects models, the effect stays robust and consistent. It thus appears that, for appropriation artists in the treatment group, trade shifted toward non-U.S. auction houses after 2013, when compared to similar artists and their artworks on sale in auction houses outside the U.S.

As we cannot fully rule out the possibility that we observe a different set of artists in pre- and post-periods,^26^ we also calculate the differences-in-differences estimator based on the same exact sample of artists as before, i.e., 1949 appropriation and control artist in total. The coefficient stays robust and negative for the reported OLS model (1) with no artist fixed-effects, and once we include the superstar-triple interaction (not reported). We thus interpret estimates as a significant reduction and relocation of around 3% points in the share of the U.S. in total auctions among treated artists after the Second Circuit decision. Again, a simple back-of-the-envelope calculation reveals that this relocation shift equals an approximate annual auction market value of 29.4 m. US-Dollars. Again, to put estimates into context, our data suggest that total auction sales range between 1.46 b. and 2.36 b. US-Dollars from 2014 to 2019.

**Table 7:** Baseline results: percentage of U.S. auctions

DV: % of U.S. auctions	(1)	(2)	(3)	(4)	(5)	(6)
	OLS	MWFE				Timeframe
Treat $\times$ post	−0.0385**	−0.0306*	−0.0335**	−0.0335***	−0.0335***	−0.0416***
	(−2.58)	(−2.40)	(−2.85)	(−7.75)	(−11.91)	(−4.42)
Year FE	$\surd$	$\surd$	$\surd$	$\surd$	$\surd$	$\surd$
Country FE	No	$\surd$	$\surd$	$\surd$	$\surd$	$\surd$
Artist FE	No	No	$\surd$	$\surd$	$\surd$	$\surd$
Cluster SE	Artist	Artist	Artist	Country	CreationY	Artist
Timeframe	2010–2020	2010–2020	2010–2020	2010–2020	2010–2020	1983–2020
*N*	168,870	168,870	168,543	168,543	168,543	257,161
${R}^2$	0.0978	0.360	0.883	0.883	0.883	0.862

*t* statistics in parentheses

*$$p<0.05$$, **$$p<0.01$$, ***$$p<0.001$$

This table shows the regression results for the dependent variable ‘percentage of U.S. auctions’ (yearly average). All results are calculated as specified above. All models include artwork-specific controls (age at auction, size, medium) and auction-house-specific controls (Phillips, Sotheby’s, Christie’s, Bonhams)

In an alternative, second identification strategy, we modify treatment and control groups and limit the treatment to appropriation artists and their auctions located in U.S. houses. Arguably, auctions by appropriation artists in different jurisdictions might have been affected differently by the changes in the legal framework. We thus consider the auctions of appropriation artists in the U.S. as treated as compared to auctions of *the same group of artists* auctioned in non-U.S. houses. Although the prominent appellate decision in the case of the visual arts has attracted broad attention in the Appropriation Art scene, in this second strategy, we hypothesize that the trade response is limited to or most impacted auction houses in the U.S. within this particular group of artists.

Accordingly, Table 8 shows regression results for the dependent variable ‘lot not sold.’ Both models are calculated with artwork- and auction-house-specific controls as in previous estimation models. And, we include year-, artist- and country-fixed effects and cluster standard errors at the artist-level (model 1) and country-level (model 2). The results for the dependent variable ‘lot not sold’ reveal that U.S. appropriation-art auctions (as compared to non-U.S. ones) faced a lower probability of sales success post-2013, with a coefficient of 0.046. This effect is statistically significant at the 1%-level. This could well reflect the short-term demand shock and the changes in buyers/sellers’ perceived litigation risks after the appellate decision in 2013. Accordingly, the decision may have affected U.S. auctions somewhat differently than those located in other countries.

As large auction houses with U.S. headquarters may operate on multiple sites and across jurisdictions, subsidiaries outside the U.S. could also have been exposed to legal changes and increased uncertainty. Anecdotal evidence suggests that Sotheby’s salesrooms in London, Hong Kong, and Dubai do not seem to engage in regulatory arbitrage. They are also governed by the laws of the state of New York as houses tend to choose the toughest laws to govern transactions which can enhance their international reputation (Shortland & Shortland, 2020). For this reason, we rerun alternative specifications inserting a dummy variable that identifies auctions located abroad but governed under the auspices of a U.S. headquarter (results not shown). Still, our main findings continue to hold.

**Table 8:** Alternative empirical strategy: appropriation market shift

DV: lot not sold	(1)	(2)
	MWFE	
$U.S.Auction \times post$	0.0460***	0.0460***
	(3.87)	(5.23)
Year FE	$\surd$	$\surd$
Country FE	$\surd$	$\surd$
Artist FE	$\surd$	$\surd$
Cluster SE	Artist	Country
Timeframe	2011–2015	2011–2015
*N*	38,346	38,346
${R}^2$	0.101	0.101

*t* statistics in parentheses

*$$p<0.05$$, **$$p<0.01$$, ***$$p<0.001$$

This table shows regression results for the dependent variable as defined in the baseline models. Samples are restricted to appropriation artists. All models are calculated with artwork-specific controls (age at auction, size, medium) and auction-house-specific controls (Phillips, Sotheby’s, Christie’s, Bonhams)

### Effect heterogeneity

So far, we have treated appropriation artists as a homogeneous group. However, the Artsy data also allow us to further distinguish and consider more fine-grained information on other closely related genomes to Appropriation Art practices, i.e., genomes other than the unique appropriation genome, for each artist in the treated group. This allows us to next address plausible heterogeneity in the treatment effect. The distribution of genomes among the appropriation artists is presented in descriptive Table 1. Based on (Artsy, 2019), we consider a set of eight related genomes for appropriation artists and interact these with the post-2013 period dummy to obtain ATTs for the different subgroups of the treated artists.

**Table 9:** Baseline results: heterogeneity in the treatment effect

	(1)	(2)	(3)
	Number of auctions	Lot not sold	% U.S. auctions
MixedMedia $\times$ post	−2.096	−0.0108	−0.0272
	(−0.03)	(−0.60)	(−1.72)
Contemporary conceptualism $\times$ post	−92.15	0.0110	−0.0269
	(−1.93)	(0.49)	(−1.58)
Engagement with mass media $\times$ post	−29.40	−0.00684	−0.0114
	(−0.50)	(−0.49)	(−1.24)
Layered images $\times$ post	−82.61	0.0298	−0.0238
	(−1.79)	(1.09)	(−1.82)
Neo-conceptualism $\times$ post	−52.79	0.0718	−0.0646**
	(−1.02)	(1.05)	(−2.72)
Photographic source $\times$ post	58.37	0.00423	−0.0349***
	(1.05)	(0.23)	(−4.75)
The pictures generation $\times$ post	−76.94*	0.0692**	−0.0565**
	(−2.24)	(3.16)	(−2.65)
Use Of vintage imagery $\times$ post	−12.90	−0.000394	−0.0417
	(−0.18)	(−0.01)	(−1.96)
Year FE	$\surd$	$\surd$	$\surd$
Country FE	$\surd$	$\surd$	$\surd$
Artist FE	$\surd$	$\surd$	$\surd$
Cluster SE	Artist	Artist	Artist
Timeframe	2010–2020	2011–2015	2010–2020
*N*	168,543	74,958	168,543
${R}^2$	0.977	0.0946	0.884

*t* statistics in parentheses, *$p<0.05$, **$p<0.01$, ***$p<0.001$

Note: This table shows the regression results for the heterogeneity in the treatment effect for the three dependent variables of the baseline results. All models are calculated with artwork-specific controls (age at auction, size, medium) and auction-house-specific controls (Phillips, Sotheby’s, Christie’s, Bonhams)

Table 9 reports model estimates for heterogeneous treatments. All models (1) to (3) replicate baseline specifications from previous sections. We run regressions on the most demanding models based on clustered standard errors at the artist level, including artist-, year- and country-fixed-effects. Again, model (1) accounts for the number of auctions and shows that all but one subgroup with related genomes are negatively affected by the court decision in 2013. The negative effect is largest for those artists that, next to the appropriation genome, are also categorized as/record the *Contemporary*-*Conceptualism* genome in the Artsy data. They see a loss of up to an average 92 auctions in the post-2013 period. Artists also associated with the group of *Photographic*-*Source* are the clear exception as they are positively affected by holding this genome, i.e., their auctions increase by 58, even though the effect renders statistically insignificant. In a similar vein, estimates for heterogeneous treatments in model (2) using ‘lot not sold’ as an outcome render mostly insignificant, and they do not always show the expected positive sign among all treated groups of artists. The heterogeneity of effects among treated appropriation artists is less pronounced when taking a look at the percentage of auctions located in the U.S. (model 3). Notably, estimated effects in each of these subcategories stay robust and negative. In addition, the heterogeneity in the treatment effects shows one common feature which we highlight in the next paragraph.

The pictures generation was an important, if not *the* most important U.S. movement of appropriation artists. It most prominently featured artists like Richard Prince, Cindy Sherman, and Louise Lawler, among several others,^27^ as outlined in the introduction Sect. 1. For this more narrowly defined group of appropriation artists,^28^, the overall perceived litigation risk around this closed group of ‘art historic’ peer-artists might be higher and, consequently, secondary art market responses could be stronger. This robustness check yields very similar and also more pronounced effects. Results for all three outcome variables confirm the overall direction of effects from baseline results and show coefficients are statistically significant throughout all models. Interestingly, relocation effects and the relative drop in the U.S. auction share are more pronounced for this group of treated artists, showing a coefficient of minus 0.056 as compared to the group of similar artists (and recalling baseline results of minus 0.0335)^29^. Moreover, the same applies to the probability that listed auctions do not sell, which brings up a positive coefficient of 0.069 (as compared to the baseline results of 0.0176). In sum, this section highlights that there is certain heterogeneity in treatment effects across models, but our main findings continue to hold for the different groups of appropriation artists as well as for a more narrowly defined sample of treated artists.

### Robustness

In this section, we test the robustness of our baseline results to possible violations of the common-pre-trends assumption, which is central to differences-in-differences models. We rerun our preferred specifications of the models with several amendments in timing, pre-periods, and pre-trends. We present the main results from these more demanding tests in the following paragraphs. Notably, we include further pre-periods to analyze potential pre-trends in the two groups of artists.

Table 10 reports a first robustness check based on a placebo timing test. Based on the same set of control and dependent variables from our baseline models, we re-estimate models (1) to (3), however, with a placebo timing that is one year prior to the 2013 court decision and under a narrow observation period (2010–2013) to again rule out initial pre-treatment noise and financial crisis confounders. If our treatment and control groups follow similar trends in dependent variables before the 2013 changes, we should observe effects that are close to zero. Model (1) shows indeed a small mean effect size of minus 3 auctions that an appropriation artist received post-2012 when compared to the control group, which is close to zero (as compared to the minus 63 to 67 auctions observed in our baseline estimates). This indicates that we do not have a general trend driving our baseline results. Similar holds for the percentage of U.S. auctions compared to non-U.S. ones and sales success probabilities. For the former (2), the coefficient $treat \times placebo timing 2012$ renders positive and insignificant (as compared to the negative significant effect in baseline estimates).^30^ For the latter (3), we obtain a very small-sized effect that is statistically insignificant (as compared to the positive effect in baseline estimates). In general, these results, together with additional robustness checks,^31^ provides very solid results and allows for a more clear-cut interpretation of the common-trends assumptions and placebo tests. Again, a notable exception is the positive 2012 coefficient for the ‘percentage of U.S. auctions’ outcome. Here, we can observe an increase in the share of U.S. auctions before the court decision. We will further address this potential issue in the event study design we discuss next.

**Table 10:** Robustness check: placebo timing 2012

	(1)	(2)	(3)
	Number of auctions	% U.S. auctions	Lot not sold
Treat $\times$ post-2012	−3.655	0.0162	0.00570
	(−1.08)	(1.75)	(0.41)
Year FE	$\surd$	$\surd$	$\surd$
Country FE	$\surd$	$\surd$	$\surd$
Artist FE	$\surd$	$\surd$	$\surd$
Cluster SE	Artist	Artist	Artist
Timeframe	2010–2013	2010–2013	2010–2013
*N*	54,333	54,333	54,333
${R}^2$	0.994	0.916	0.0986

*t* statistics in parentheses

*$$p<0.05$$, **$$p<0.01$$, ***$$p<0.001$$

This table shows the placebo-timing regression results for the three dependent variables as defined in the baseline models. All models are based on MWFE regressions with artist-, year, and country-fixed effects and standard errors clustered at the artist level. All models are calculated with artwork-specific controls (age at auction, size, medium) and auction-house-specific controls (Phillips, Sotheby’s, Christie’s, Bonhams). Models include a superstar $\times$ treat $\times$ post-triple-interaction (not reported)

Building on the previous literature on differences-in-differences models (Autor, 2003; Roth, 2019), we next inspect if there is a significant difference (and trends) in outcome variables in periods before the treatment.

**Fig. 7:**
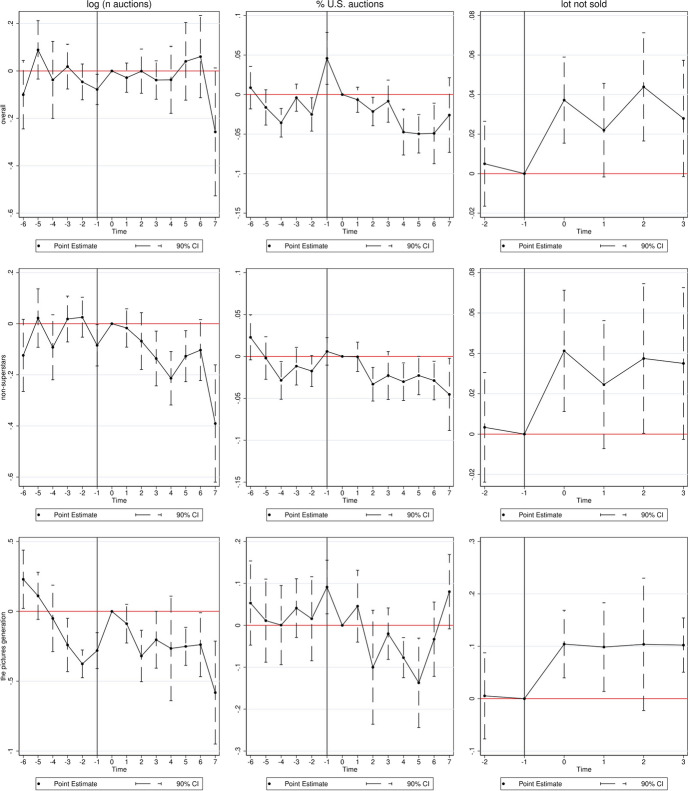
Event Study Analysis. *Note* This figure shows the event study estimates for the coefficient of interest computed based on the baseline results. The three panels in the left column for the ‘(log) number of auctions,’ middle column the ‘percentage of U.S. auctions,’ and right column ‘lot not sold.’ Top raw is based on the overall sample, mid raw based on the non-superstar sample, and the bottom raw computed using only ‘the pictures generation’ as treated units. We show point estimates and 90% confidence intervals. Coefficients are normalized around the base year 2013 (2012 for ‘lot not sold’). All models include artist, auction year, artwork, auction country, and auction house controls. Standard errors are clustered at the artist level

The event study results are presented and visualized in Fig. 7. We construct the event study with the (non-staggered) model by regressing on leads and lags with the post-event time ($t>2013$), for the appropriation artists as treated units, and similar artists serving as pure controls. All models include the most demanding specification using artist, artwork, auction-country, auction year and auction-house controls. Standard errors are clustered at the artist-level in models. The three panels in the left column show the ‘(log) number of auctions,’ mid column the ‘percentage of U.S. auctions,’ and right column ‘lot not sold.’ For the sake of transparency, we report event study estimates for different samples. Top row panels are based on the overall sample, mid-row ones are based on the non-superstar sample, and the bottom row panels are computed using ‘the pictures generation’ as treated units only.

As argued above, estimated results for the log-transformed number of auctions in the first column are mixed. While the overall effect renders insignificant, estimates for the non-superstars confirm a valid pre-trend with close to zero coefficients for the years prior to the court decision. As coefficient estimates for lags indicate, treated units (appropriation artists) show a constant and significant negative decline in the yearly number of auctions after the verdict. This is important to note. Moreover, sample estimates for ‘the pictures generation’ as treated artists also show an overall negative trend for post-2013 outcomes, albeit a negative pre-trend for years prior to the court decision must be acknowledged. Hence, we can only cautiously interpret the treatment as causal for the most demanding models, as some estimates indicate the presence of pre-trends for the ‘most treated’ group of artists.

At large, we observe heterogeneous treatment effects on the share of U.S. auctions. Still, it must be noted that estimated effects render increasingly negative in post-periods across all samples (mid-column panels in Fig. 7). Arguably, as the placebo-timing test model (2) in Table 10 indicated, the increase in the percentage of U.S. auctions for the treatment group before treatment time ($t_0$) could balance out the significance of our differences-in-differences results obtained in the baseline model (Table 7). In principle, this could justify the weakly significant pre-trends we observe in estimates. At large, however, results for the non-superstars and ‘the pictures generation’ samples support the general pre-trend assumption and show consistently lower estimated coefficients throughout post-periods. Although only two out of four coefficient estimates (leads) in the overall sample render insignificant and yield a close-to-zero effect in pre-periods, there is a clear common trend prior to the treatment observed for the non-superstars sample. As pre-trends in the most demanding specifications might effectively downward-bias the average treatment effect on the treated, we again cautiously interpret the coefficient of interest for this outcome variable as causal.

Finally, the right-hand column in Fig. 7 presents event study estimates for the outcome variable ‘lot not sold.’ Here, we can again observe a clear-cut zero effect prior to the court decision and a sudden upward jump in unsold auction probabilities for the treatment group in the first 3 years after the verdict.^32^ Two out of four post-period coefficients (lags) show the expected statistically significant effects in the overall sample, while we observe insignificant estimates on slightly lower absolute levels for the non-superstar panel. Notably, estimates for ‘the pictures generation’ again show a very consistent pattern of significant estimated post-coefficients compared to pre-periods. As the common pre-trends assumption is supported for all lot-not-sold models, we interpret the differences-in-differences results for this outcome variable as causal.

While general robustness checks and event study results in particular, point toward a certain heterogeneity in the overall data sample, we are confident that main results and causal research claims continue to hold. In the next section, we discuss the policy implications of our findings.

## Policy discussion

The research indicates that doctrinal complexity and legal uncertainty around U.S. fair use in the visual arts increased following the 2013 court decision. In this particular case, changes might not have helped to promote economic efficiency around licensing in the U.S. (as intended by the fair use doctrine) because the criteria to apply fair use in the first place seemed less clear and laws seemed less predictable after the decision [Landes (2000), Adler (2016)]. In this way, perceived litigation risks for artists and trade intermediaries have increased in due course.

We provide quantitative evidence on the costs and market charges for imposing indirect liability for infringement on intermediaries in the visual arts. Once their perceived litigation risk increases, this will affect the curation of works and selection into sales. Arguably, however, this research does not provide a comparative assessment of the effectiveness of indirect liability vis-á-vis other types of direct rights enforcement or changes in copyright’s scope or length that would also modify incentives to reuse and disseminate artworks. And, it is not clear how much results generalize to other sectors of the creative economy. There is likely a distinction to be made between the criteria set up by automated filtering of millions of titles as in online music (for example, YouTube’s content id system) and the manual selection and expert curation of a limited number of works for sale as in the visual arts. Even when copyright decisions, as we assume, do not cause creative practices by (appropriation) artists themselves to change, based on our findings, contributory liability and changes in perceived litigation risks temporarily limit the marketplace and auction showrooms made available to these artists because the relative sales value of their artwork (as compared to other non-infringing artwork) changes in due course. In the longer term, we would expect investment and growth of art market segments that involve follow-on innovation and that bear higher infringement risk to be restricted, if the nature of fair use is not evolving with new jurisprudence and risk perceptions are again changing.

Results also corroborate the idea that countries are competing over national legal frameworks and that firms and services are responding to the overall legal climate set up by national jurisprudence. Where laws and legal practices are not harmonized on an international level, intermediaries on global visual art markets such as auction houses will tend to migrate mobile ‘factors of production/services’ to those jurisdictions that offer the most favorable conditions to them. This need not always be the case in visual art markets as evidence from previous research on resale rights suggests Banternghansa and Graddy (2009). Still, for the Appropriation Art case under scrutiny here, some sales value has shifted to places outside the U.S., with seemingly lower perceived litigation risks for auction houses and different sets of copyright rules in force.

In general, copyright protection not only ring-fences creators from unauthorized copying of others. Beyond the cases of authorized reuse and licensing, as we have shown with the data and extensive analysis, temporary uncertainty around fair use rules also restricts artists’ unauthorized follow-on innovation in the context of the visual arts, i.e., ‘transformative’ and ‘productive’ reuses of original artworks. While unauthorized (reproductive) copying seems much less of an issue in the visual arts, follow-on innovation is a growing policy concern with the changes and assemblage using new digital technologies and reuses of original artworks that are (also) protected by copyright (cf. Table 9). In this way, copyright rules also strike a delicate balance between new and older generations of artists and give them more or less freedom to operate and room to develop new reusing art practices in due course. The latter topic was not part of this investigation, but is an open issue and left to future research.

## Conclusion

In this research, we show that there is a role for copyright in the visual arts, in particular in cases of follow-on innovation and in the appropriating arts, beyond the basic control over copying and reproductive uses. We investigate how the prominent 2013 *Cariou v. Prince* U.S. court decision affected trade and availability in this sector. This decision arguably called into question prior assumptions about the application of fair use for some types of visual artworks and may have increased legal uncertainty concerning those works for market intermediaries. From an art historic perspective, results indicate that past legal framework changes have affected trade and relative market value in the appropriating arts. So, eventually, rules have also impacted the general direction of genre development and innovative practices in the figurative arts.

More specifically, quantitative findings on intermediary liability suggest that global auction trade in Appropriation Art, at least temporarily, decreases and partially relocates to other, non-U.S. jurisdictions following the fair use decision. Moreover, for artworks listed in auctions, the sales probability of potentially infringing (appropriating) artworks decreases in this period. Effects are most pronounced for treated artists from ‘the Pictures Generation,’ an art movement that has pioneered appropriation techniques. Moreover, effects on auction trade continue to hold once we control for superstar artists, which, arguably, might have higher general visibility in markets, higher value in court disputes, and, accordingly, impose higher liability risk on traders.

At large, back-of-the-envelope calculations reveal an estimated global market value of around 290–304 m. U.S. Dollars forgone due to fewer artworks auctioned after the decision, while the total yearly auction sales volume, based on our data, ranges between 1.46 b. and 2.36 b. US-Dollars from 2014 to 2019. We interpret and relate our findings to a temporary increase in the perceived litigation risks in the visual art environment, in particular in terms of the contributory liability of intermediaries and the charges on global artwork trade by auction houses. Findings are robust against several alternative specifications and placebo testing.

## Footnotes

1 For example, Watt et al. (2014) develop economic theory in the visual arts describing how copyright can help artists exploit their works by creating a secondary market for copies and reproductions.

2 The concept of copying and using existing materials in art has evolved over time. Early 20th-century avant-garde artists like Pablo Picasso incorporated objects from other artists into their works and collages as an archetypal conceptual innovation (Galenson, 2009), while Marcel Duchamp repositioned ordinary objects (ready-mades) in the museum setting. The 1960s Pop Art movement saw the reuse of advertising materials and famous images like the Mona Lisa. Contemporary artists from the Pictures Generation like Sherrie Levine, Elaine Sturtevant, Louise Lawler, Cindy Sherman or Richard Prince have raised generic questions about originality and authenticity through their use of existing art in new works. For example, the famous *Cowboy Series* is emblematic of the artwork by Prince, where he rephotographed Marlboro’s (older) advertisements (Phillips, 2021). Sturtevant reproduced artworks by contemporary artists which were visually indistinguishable from the originals, probably best known for her series titled *Warhol Flowers* (Artnet, 2021). In a similar vein, Levine photographed reproductions of the male photographer Walker Evans (titled *After Walker Evans*) and replicas of Duchamp’s Urinal titled *After Marcel Duchamp*, partially because she wanted to highlight how female voices were undervalued in the art world (MET, 2021; Tate, 2021). Other forms of Appropriation Art practices include détournement, pastiche, rephotography, and parody (Evans, 2009).

3 In a similar vein, according to (Landes & Lichtman, 2003), adjustments to the scope and duration of copyright protection can provide an alternative approach to imposing indirect liability because that also tailors the incentives to create and disseminate works. In general, there is vast empirical literature on the effects of different types of interventions and enforcement rules on the levels of infringement online and offline which due to space constraints we will not cover here. For an overview, see Danaher et al. (2017).

4 That said, e.g., visual artists rights associations and registries offer support for licensing to those who want to reproduce works of visual art, mostly reproductions of their artwork in, e.g., books, postcards or merchandise (Posner, 2004).

5 Among early Appropriation Art cases, in 1992 *Rogers v. Koons*, Koons was sued for transforming a photograph into a sculpture. Based on fair use, Koons argued that his sculpture should be privileged as a satirical comment or parody, but the court rejected this (Ames, 1993; Landes, 2000; Posner, 1992). Already at that time, the decision was not well received by artists because of their fear this might cripple Appropriation Art (Landes, 2000). The same artist, Koons, won the next case in 2006 *(Bloom v. Koons)* where the court held that the reusing work differed sufficiently from the original. For a detailed review of copyright fair use cases in the visual arts and the road to *Cariou v. Prince* see, e.g., Adler (2016). A more recent case is the *Warhol v. Goldsmith* decision from early 2021, accessible here.

6 Excerpt from Cariou v. Prince, 784 F. Supp. 2d 337, 98 U.S.P.Q.2d 1318 (S.D.N.Y. 2011).

7 Some further voices from the art scene are represented in ArtNews (2014), among others, the Andy Warhol Foundation and Amy Goldrich, which framed the decision as ‘one of the most significant art copyright cases of recent decades’ [...] and further argued that it was a positive outcome for appropriation artists and ‘those whose work may be sources for appropriation will likely be disappointed by this outcome.’ We also provide further evidence on the importance of the exogenous shock by analyzing google online searches in Sect. 3.3.

8 After initial registration, artist meta-data from the Artsy website can be accessed via the public application programming interface (API). Python code used in the paper will be provided online.

9 According to (Artsy, 2019) close ‘genomes’ of ‘Appropriation Art’ are: contemporary conceptualism, engagement with mass media, layered images, mixed media, neo-conceptualism, photographic source, the pictures generation, use of vintage imaginary.

10 Artsy’s similar artists feature ‘continuously computes a K-nearest neighbor graph for artists using data from the Art-Genome-Project’ (Artsy, 2020). The 1,000+ characteristics of artists include art historical movements, subject matter, and formal qualities, a detailed list is provided here (Artsy, 2021). We manually double-checked the ‘appropriation’ classification by comparing the queries with known and listed artists on, e.g., Wikipedia, (Tate, 2021) or (Evans, 2009). Chapter 3.2 furthermore describes differences between appropriation artists and similar artists on the artist-level, and chapter 3.3 compares outcomes (auction results) in both groups to make sure that the groups are sufficiently close based on objective criteria.

11 40% of unmatched appropriation artists are born in the U.S., followed by many British, Chinese, and French-born artists. However, we observe fewer Chinese-born appropriation artists in matched samples. Roughly 33 (37) % of unmatched artists are female, and, on average, artists are 12 (7) years younger compared to matches in the appropriation sample (control group). Arguably, this suggests that some younger artists and recent artworks have not yet entered secondary markets, and thus, their actual market values might be less well covered in both samples.

12 The economic literature on art auctions also discusses the so-called ‘master-piece effect’ (Ashenfelter & Graddy, 2003) which relates returns from investment in paintings of well-known artists to those garnered from investment in less-known, second-tier artwork. The empirical evidence is ambiguous so far, ranging from a positive effect (De la Barre et al., 1994) to no or a negative effect of more investment in masterpieces (Ashenfelter & Graddy, 2003; Ginsburgh & Jeanfils, 1995; Mei & Moses, 2001; Pesando, 1993).

13 Thus, artwork with certain artwork titles, i.e., ‘sans titre,’ ‘untitled,’ etc., is excluded from samples. If artworks record multiple years of creation, the first year is selected

14 The highest percentage of artists in the control group mostly originate from the U.S. (26%), Great Britain (7%), Germany (6%) and France (4%). In addition, similar artists are most frequently located and working in New York (roughly 8%), London (5%), L.A. (5 percent), Paris (4%), and Berlin (3%). Notably, figures warrant careful interpretation as some artists record more than one work location/residence and because location names are not fully harmonized across the data.

15 The New York MET-collection database is the largest art museum in the U.S. with a focus on contemporary art. Based on this step, we can identify 53 artists with a total of 178 artworks collected by the MET museum, and, web-crawling their recent artworks’ exhibition history, results in a total of 702 exhibitions for them (exhibited in the MET or on loan to other museums).

16 Please refer, for instance, to the recent Appropriation Art copyright lawsuit where a work by the artist Jeff Koons has been removed from an exhibition in the Museum Pompidou in Paris (Artnet, 2017).

17 For more information, we discuss the court decision in footnote 5. While the interest in the ‘Cariou v. Prince’ decision was never outpaced by Google searches around the ‘Rogers v. Koons’ case, we interpret the overall interest in a similar court case by that time as positively correlated with the importance of ‘Cariou v. Prince’).

18 For the set of treated artists, we have information on both a tighter definition of *Appropriation Art* artists and a wider definition of contemporary artists associated with or categorized as artists applying ‘appropriation’ practices (including the appropriation-genome). Again, it is important to note that not all artworks in the group of treated artists will infringe copyright. But since not all rights around potentially copyright-infringing artworks are cleared, *potentially* infringing artists and artworks are equally important levels of the analysis to consider as the perceived risk of copyright litigation will still shift incentives to trade these artworks.

19 For example, it is common practice to publish a pre-sale catalog with information on the auction items up for sale and houses are typically commissioning the work for sellers entering a contractual relationship with them (consignment agreement) that cannot be immediately be canceled. For standard practices at art auctions, see Ashenfelter and Graddy (2003).

20 Based on the results from the hedonic price models, we control for the four biggest auction houses, as this effect captures most of the variation of auction house differences we found, and, furthermore, reporting practices across auction houses can vary (Ashenfelter & Graddy, 2003).

21 In particular, the same artwork can be traded in different countries and auction-houses. For example, given an artwork is titled differently or several multiple-prints of the ‘same’ artwork exist, as well as reporting standards vary between auction houses, in our large auction sample, it seems impossible to identify and manually verify the same artworks over time in our sample. Data samples used in hedonic price models are, moreover, typically hand-collected and often focus on listings of a single auction house or country, well-known artists and their catalog raisoneés, or they are restricted to a single medium (e.g., paintings).

22 Auction-specific controls include dummy variables for auctions at Phillips, Sotheby’s, Christie’s, and Bonhams. The specific shares of auction houses in total auctions are reported in Table 2. Alternative models (not reported in the table, available upon request) yield similar results when adding more or different controls for auction-specific characteristics, i.e., controls for i) more auction houses than the top ones, ii) country-specific auction houses, or iii) combinations thereof.

23 Arguably, superstars and their artworks often have higher commercial value and are more visible in markets, as descriptive statistics show. This might also ease monitoring and enforcement of predecessors’ copyrights and increase the potential value in disputes that involve superstars. So, superstars, together with their traders, might be more likely to enter infringement proceedings, and, hence, trading their artworks could impose a higher liability risk on auction houses.

24 The dummy variable ‘superstar’ is constructed as the 75th percentile of the overall panel distribution of the ‘number of auctions’ outcome variable. The dummy is one for an artist exceeding the threshold of 478 auctions, zero otherwise. Prominent artists classified in this way account for roughly one-fourth of auctions in the overall data, while the group of appropriation artists accounts for 57% of all auctions attributed to superstars. By this definition, for example, Andy Warhol, Roy Lichtenstein, Pablo Picasso, and Damien Hirst are considered superstars.

25 One notable exception is that fewer top auction houses (Philipps, Christie’s, and Sotheby’s) seem to curate and list artworks as their shares in samples decline after the decision. However, this pattern similarly applies to works by appropriation and similar artists.

26 For example, there might be a potential selection bias from similar newcomer artists in Europe, first entering the panel post-2013.

27 For an overview, please refer to this non-technical summary on Wikipedia or an article from Artnews (2017).

28 The artists from ‘The Pictures Generation’ in our sample account for 2134 auctions between 2010 and 2020, of which 61% were listed in auction houses in the U.S.

29 Note that, interestingly, ‘The Pictures Generation’ is generally associated with artists based and worked in the 1980s in the U.S. (Artnews, 2017), and 96% of the recorded auctions are indeed from artists with U.S. nationality, while the remaining 91 auctions are from the artists Jack Goldstein (Canada born) and Thomas Lawson (British), both live(d) and work(ed) in the U.S.

30 The inclusion of the triple interaction term capturing the superstar effect (not reported) seems important for the percentage share of U.S. auctions. Otherwise, the shift of the 2012 coefficient is more pronounced.

31 We also re-run our baseline models and placebo timing test by dropping 2012 observations from the sample to see whether the effects are driven by this particular year of auctions (not reported).

32 The coefficients are normalized around $t_{-1}$ given the dependent variable ‘lot not sold’ at the auction day-level.
